# Supplementary material for: Synthesis, Structural Characterisation, and Electrochemical Properties of Copper(II) Complexes with Functionalized Thiosemicarbazones Derived from 5-Acetylbarbituric Acid
Source: Molecules. 2024 May 10;29(10):2245. doi: 10.3390/molecules29102245 (PMC11124361; doi:10.3390/molecules29102245)
Supplement: Supplementary file 1 [file molecules-29-02245-s001.zip › molecules-2997287-supplementary.pdf]

Molecules

Synthesis, structural characterization and electrochemical properties of Copper(II) complexes with Functionalized Thiosemicarbazones derived from 5-Acetylbarbituric Acid

Alfonso Castiñeiras 1, Nuria Fernández-Hermida 1, Isabel García-Santos 1,*, Lourdes Gómez-Rodríguez 1, Antonio Frontera 2 and Juan Niclós-Gutiérrez 3

1 Department of Inorganic Chemistry, Faculty of Pharmacy, University of Santiago de Compostela, 15782 Santiago de Compostela, Spain; alfonso.castineiras@usc.es (A.C.); lurdesgomezrodriguez@edu.xunta.es (L.G.-R.)

2 Department of Chemistry, University of Illes Balears, Crta de Valldemossa km 7.5, 07122 Palma de Mallorca, Spain; toni.frontera@uib.es

3 Department of Inorganic Chemistry, Faculty of Pharmacy, University of Granada, 18071 Granada, Spain; jniclos@ugr.es

** Correspondence: isabel.garcia@usc.es*

Supplementary material

Table S1. Hydrogen bond parameters pag. 2

Table S2. Intermolecular ring-ring and ring-metal interaction parameters pag. 3

Figure S1. Mass spectra pag. 4

Figure S2. FT-IR spectra in the region 4000-500 cm^{-1} pag. 6

Figure S3. FT-IR spectra in the region 500-100 cm^{-1} pag. 12

Figure S4. UV-visible Spectra pag. 17

Figure S5. EPR spectra pag. 22

Figure S6. View of the unit cell for **3a** psg. 26

Figure S7. View of the unit cell for **4** pag. 27

Figure S8. View of the unit cell for **6** pag. 28

Figure S9. Cyclic voltammograms pag. 29

Table S1. Hydrogen bond parameters [Å, º] for [Cu(HAcbDM)(H_2O)$_2$](NO_3)·H_2O (**3a**), [Cu(HAcbDM)(H_2O)$_2$]ClO_4 (**4**), [Cu(HAcbHexim)Cl] (**6**). The letters in brackets refer to the symmetry codes shown in the text and figures.

Compound	D–H···A	D–H	H···A	D···A	∠DHA	Symmetry code
**3a**	O(12)-H(12A)···O(31)[a]	0.94	1.83	2.753(6)	169.5	-x+1,-y+1,-z+1
	O(12)-H(12B)···O(14)[b]	0.92	1.86	2.743(5)	160.3	-x+2,-y+1,-z+1
	O(14)-H(14A)···O(1)	0.95	2.24	2.902(6)	126.1	
	O(14)-H(14B)···O(23)[d]	0.87	1.91	2.775(5)	169.8	-x+1,-y,-z+1
	N(11)-H(11)···O(23)[e]	0.93	1.88	2.789(5)	167.0	x+1,y+1,z
	N(13)-H(13)···O(42)	0.90	2.29	3.084(7)	147.4	
	N(15)-H(15)···O(15)[f]	0.89	1.95	2.829(5	170.9	-x+2,-y+1,-z
	O(22)-H(22B)···O(41)[g]	0.95	1.70	2.646(5)	176.8	-x+1,-y,-z
	O(24)-H(24A)···O(2)	0.93	2.09	2.765(6)	128.0	
	O(24)-H(24B)···O(22)[h]	0.91	1.86	2.699(5)	152.2	-x,-y,-z
	N(21)-H(21)···O(13)[i]	0.94	1.88	2.784(5	159.2	x-1,y-1,z
	N(23)-H(23)···O(32)	1.06	2.16	3.165(8)	157.0	
	N(25)-H(25)···O(1)[d]	0.97	1.90	2.865(5)	171.4	-x+1,-y,-z+1
	O(1)-H(1A)···O(43)	1.05	1.91	2.879(7)	152.1	
	O(1)-H(1B)···O(25)[c]	1.04	1.77	2.802(6)	169.5	x+1,y,z
	O(2)-H(2B)···O(32)	0.90	2.54	3.411(9)	163.9	
**4**	O(1)-H(1A)···O(2)[a]	0.96	1.83	2.680(12)	146.1	-x+1,-y,-z+1
	O(1)-H(1B)···O(16)[b]	0.96	2.09	2.716(12)	121.7	-x+1/2,y-1/2,-z+1/2
	O(2)-H(2A)···O(13)[c]	0.96	2.01	2.856(11)	146.6	x,y,z-1
	O(2)-H(2B)···O(18A)	0.96	2.61	3.24(2)	123.5	
	O(2)-H(2B)···O(18B)	0.96	2.21	3.11(3)	156.2	
	N(11)-H(11)···O(13)[d]	0.86	2.06	2.915(13)	175.7	-x+1,-y,-z+2
	N(15)-H(15)···O(18A)[e]	0.86	2.29	3.14(2)	172.8	x,y,z+1
	N(15)-H(15)···O(19B)[e]	0.86	2.28	3.06(3)	150.4	x,y,z+1
**6**	N(11)-H(11A)···O(15)[a]	0.84(3)	2.04(3)	2.851(3)	162(3)	-x+1,y-1/2,-z+1/2
	N(13)-H(13A)···O(13)[b]	0.86(3)	2.19(3)	2.939(3)	145(3)	x-1/2,y,-z+1/2
	N(15)-H(15A)···Cl(1)[c]	0.80(3)	2.72(3)	3.437(2)	151(3)	-x+1,y+1/2,-z+1/2

Table S2. Intermolecular ring-ring and ring-metal interaction parameters (Å, º) for $[Cu(HAcbDM)(H_2O)_2]ClO_4$ (**4**)*.

**Ring**	**π···π**	**Cg-Cg**	**α**	**IPD**	**Symmetry**
Cu1/S1/C18/N13/N12	Cg1-Cg1[a]	3.7151	0	3.6077	-x,-y,1-z
Cu1/S1/C18/N13/N12<br>Cu1/O11/C11/C16/C17/N12	Cg1-Cg2[a]	3.8532	5	3.6203	-x,-y,1-z
Cu1/O11/C11/C16/C17/N12<br>Cu1/S1/C18/N13/N12	Cg2-Cg1[a]	3.8532	5	3.4833	-x,-y,1-z
Cu1/S1/C18/N13/N12	Cg1-Cu1[a]	3.911	22.51		-x,-y,1-z

*CgI/CgJ are the centroids of the corresponding rings. Cg-Cg is the center-to-center distance (distance between ring centroids), α is the angle between mean planes of the rings, IPD is the mean interplanar distance (distance from one plane to the neighboring centroid). For details, see Janiak, C. (2000). J. Chem. Soc. Dalton Trans. pp. 3885–3898.

Figure S1. Mass spectra of the complexes (FAB).

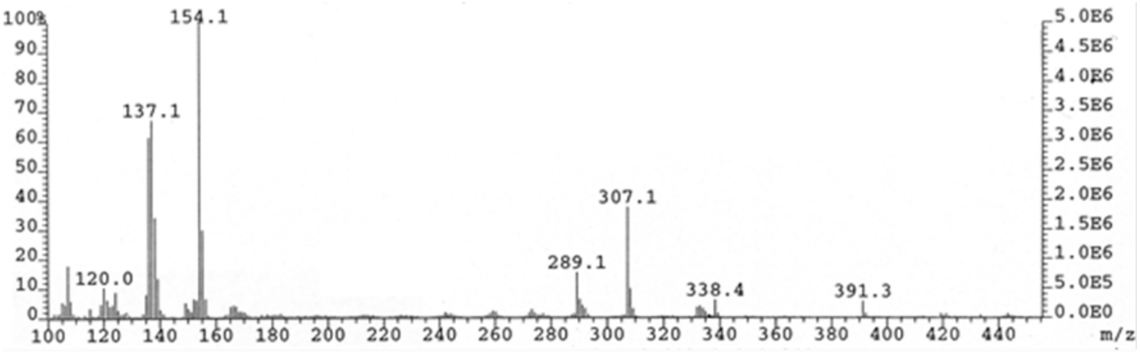

[Cu(HAcbDM)(OAc)]·3H₂O (**1**·3H₂O)

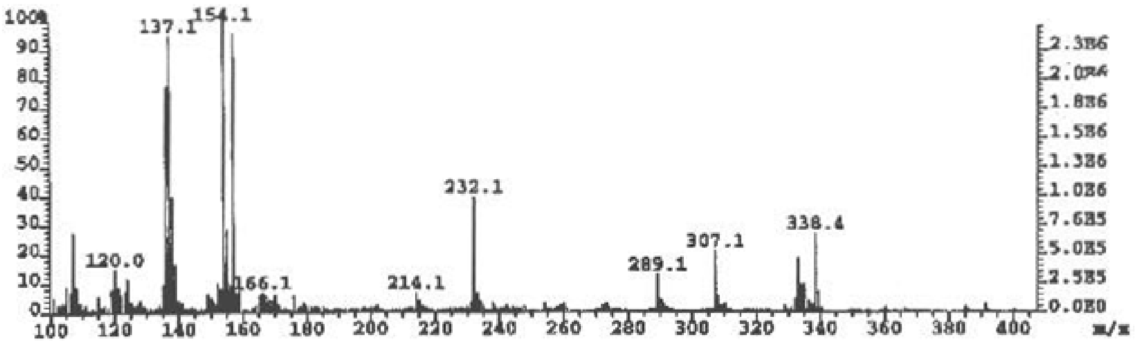

[Cu(HAcbDM)Cl]·1/2EtOH (**2**·1/2EtOH)

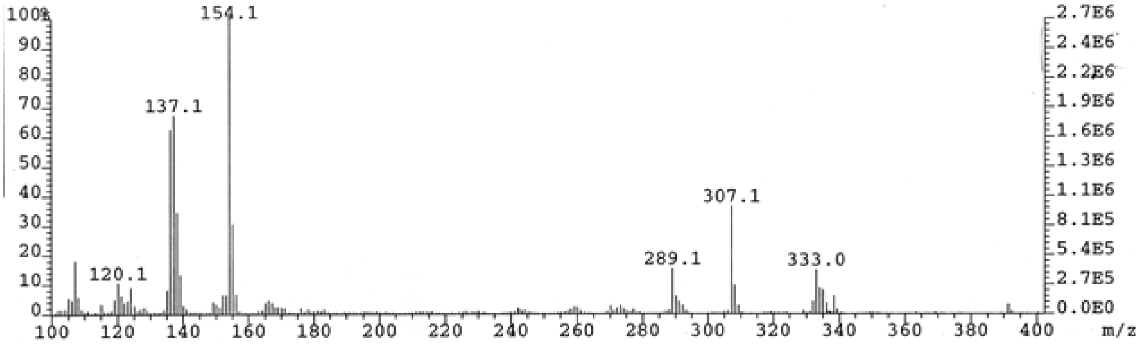

[Cu(HAcbDM)(NO₃)] (**3**)

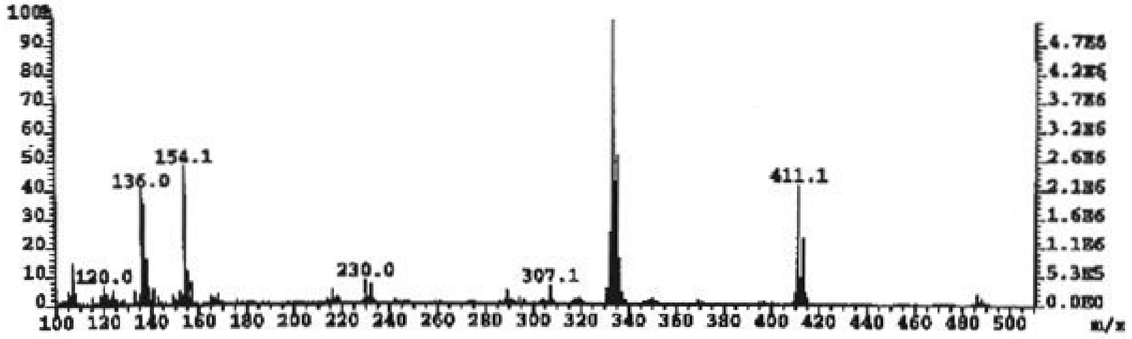

[Cu(HAcbDM)(H₂O)₂]ClO₄ (**4**)

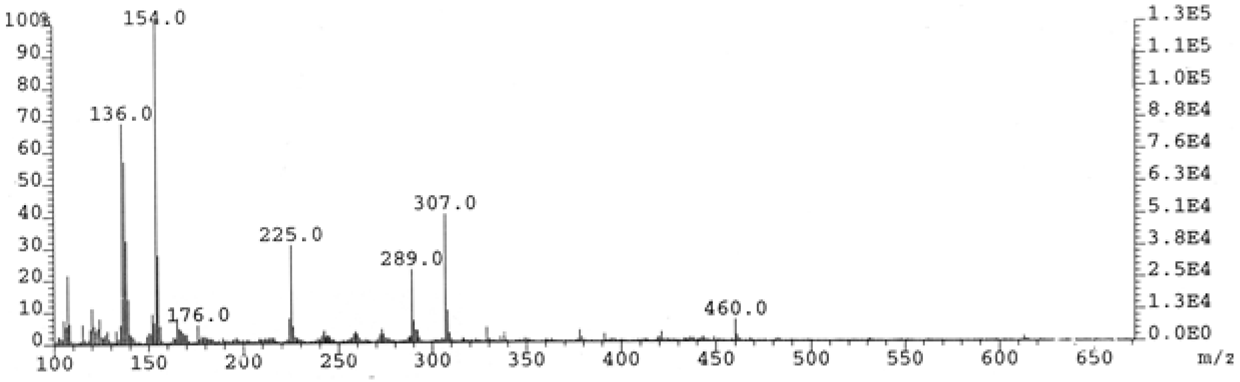

[Cu(HAcbHexim)(OAc)]·5H₂O (**5**·5H₂O)

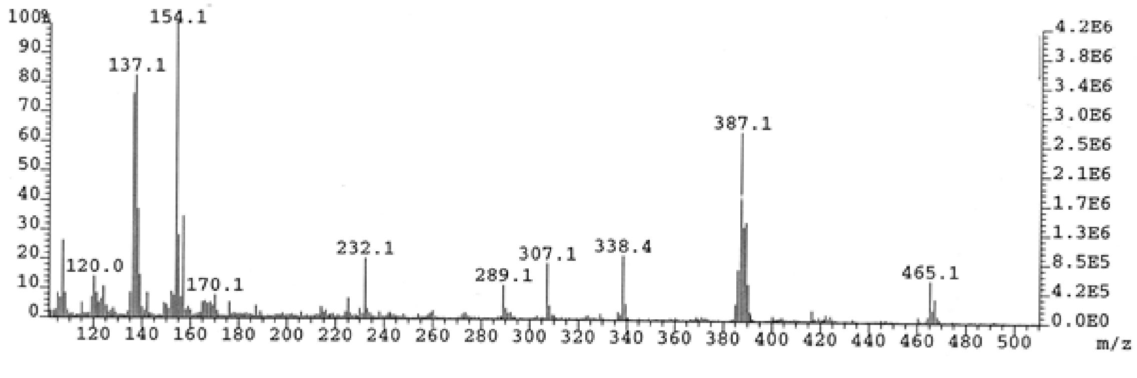

[Cu(HAcbHexim)Cl]·1/2EtOH (**6**·1/2EtOH)

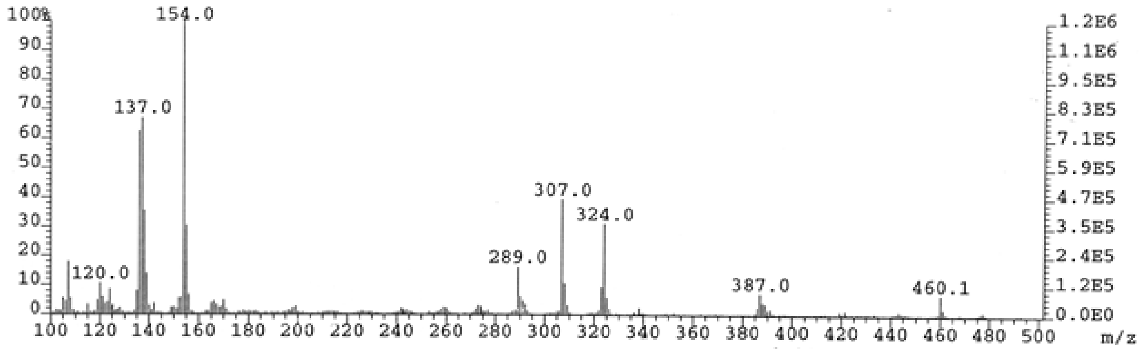

[Cu(HAcbHexim)(NO$_3$*)]·1/2H*$_2$*O* (**7**·1/2H_2O)

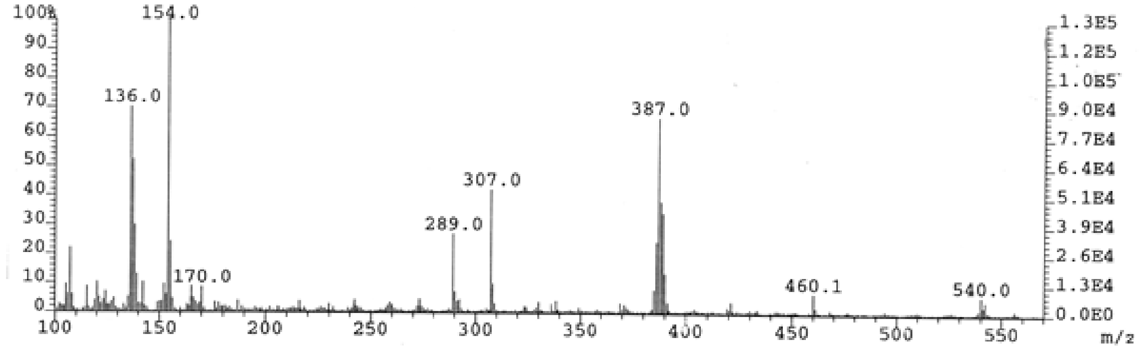

[Cu(HAcbHexim)ClO$_4$*]* (**8**)

Figure S2. FT-IR spectra in the region 4000-500 cm⁻¹

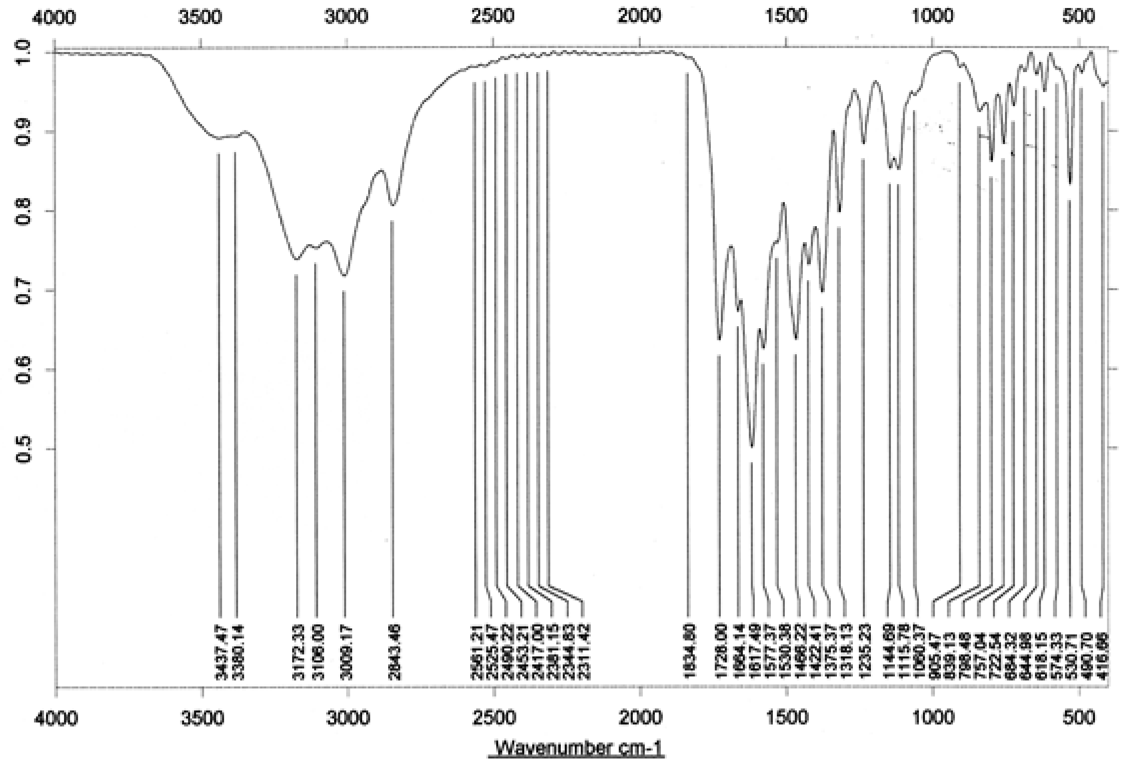

$H_2AcbDM{\cdot}H_2O$

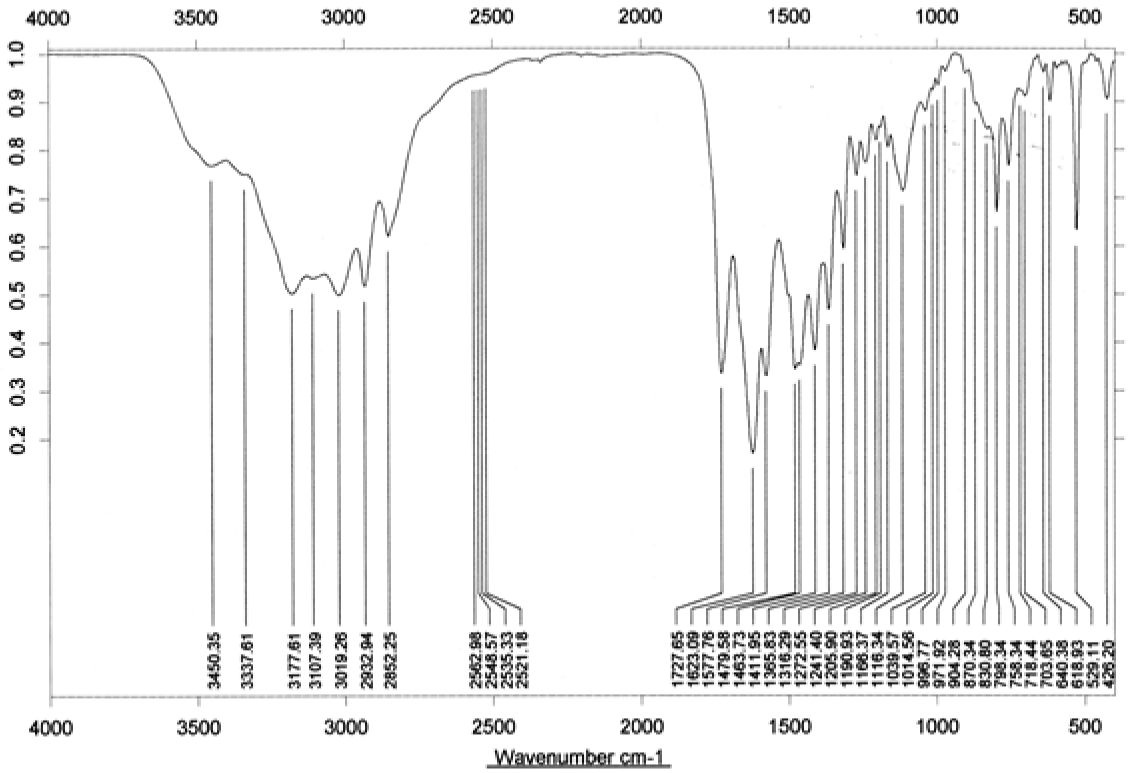

$H_2AcbHexim{\cdot}H_2O$

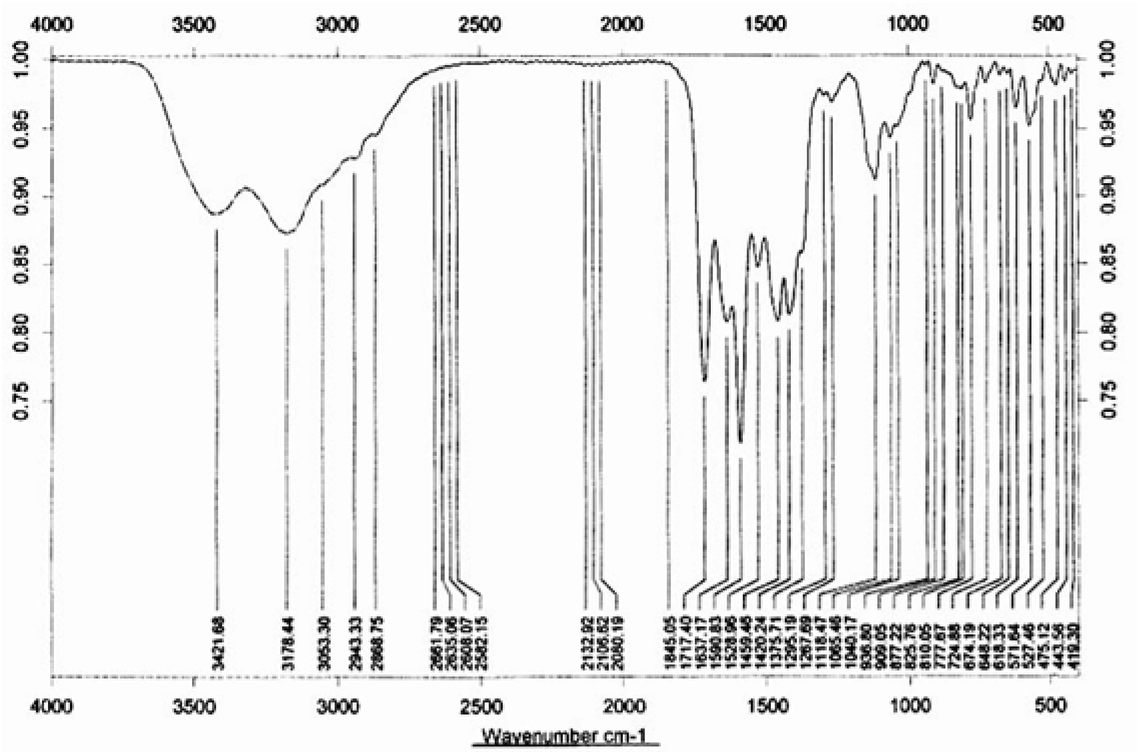

[Cu(HAcbDM)(OAc)]·3H₂O

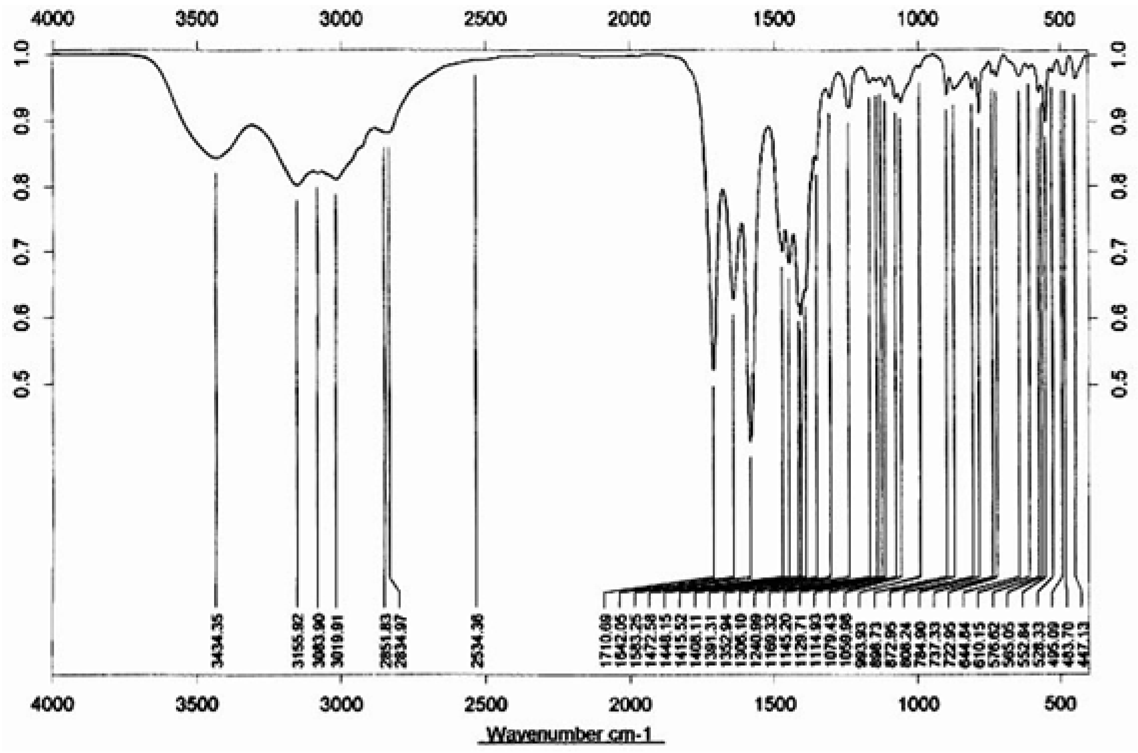

[Cu(HAcbDM)Cl]·1/2EtOH

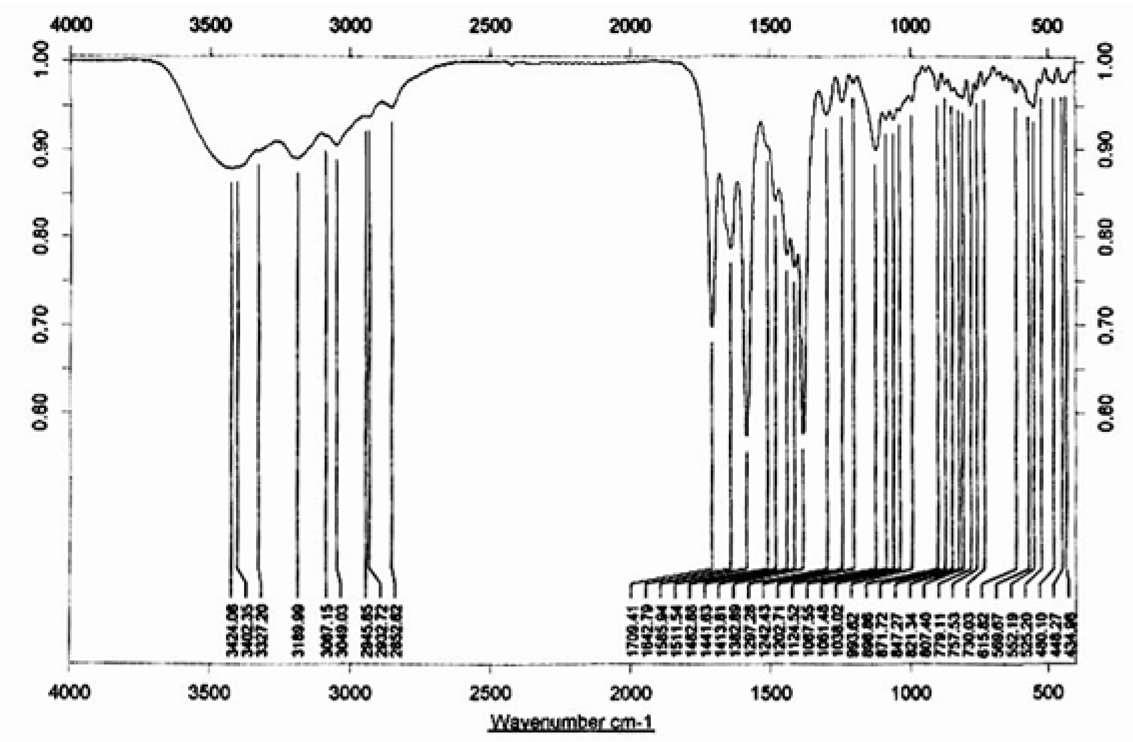

[Cu(HAcbDM)(NO₃)]

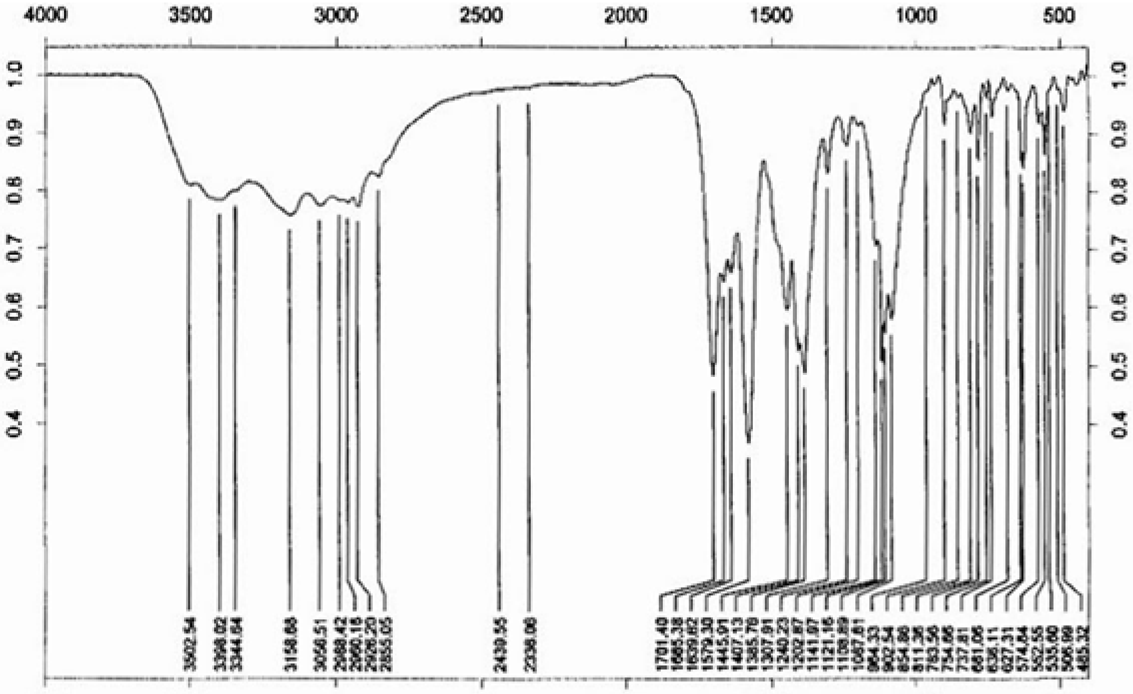

$[Cu(HAcbDM)(H_2O)_2]ClO_4$

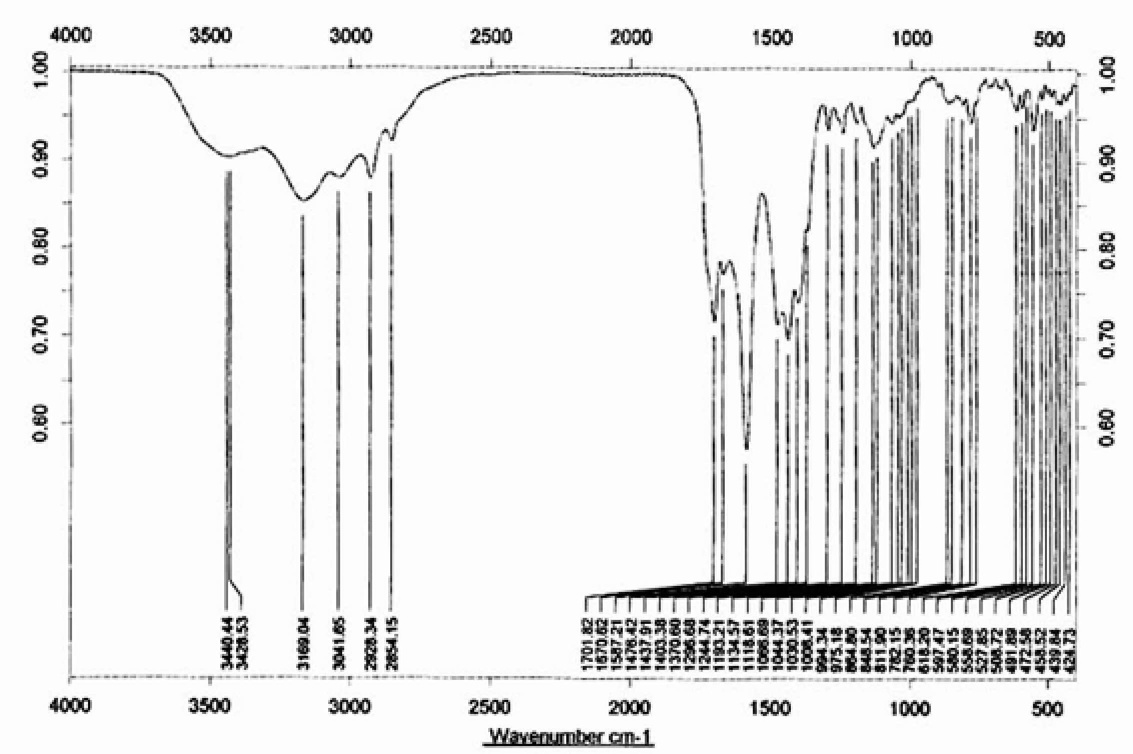

[Cu(HAcbHexim)(OAc)]·5H_2O

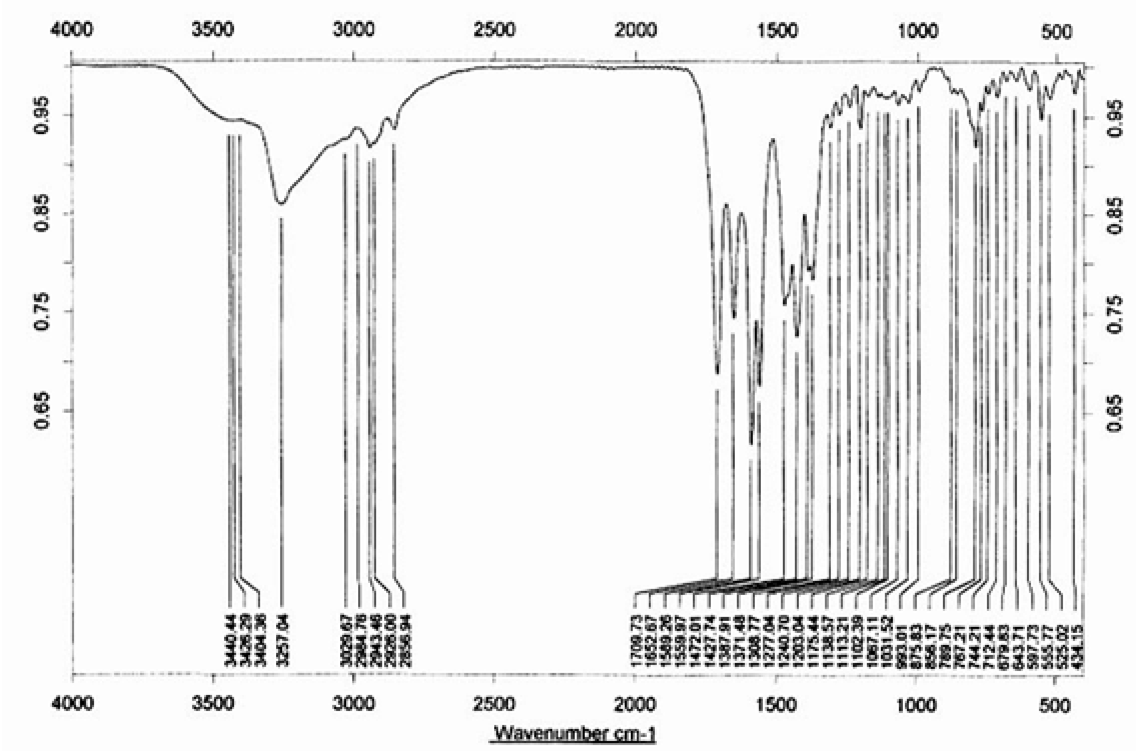

[Cu(HAcbHexim)Cl]·1/2EtOH

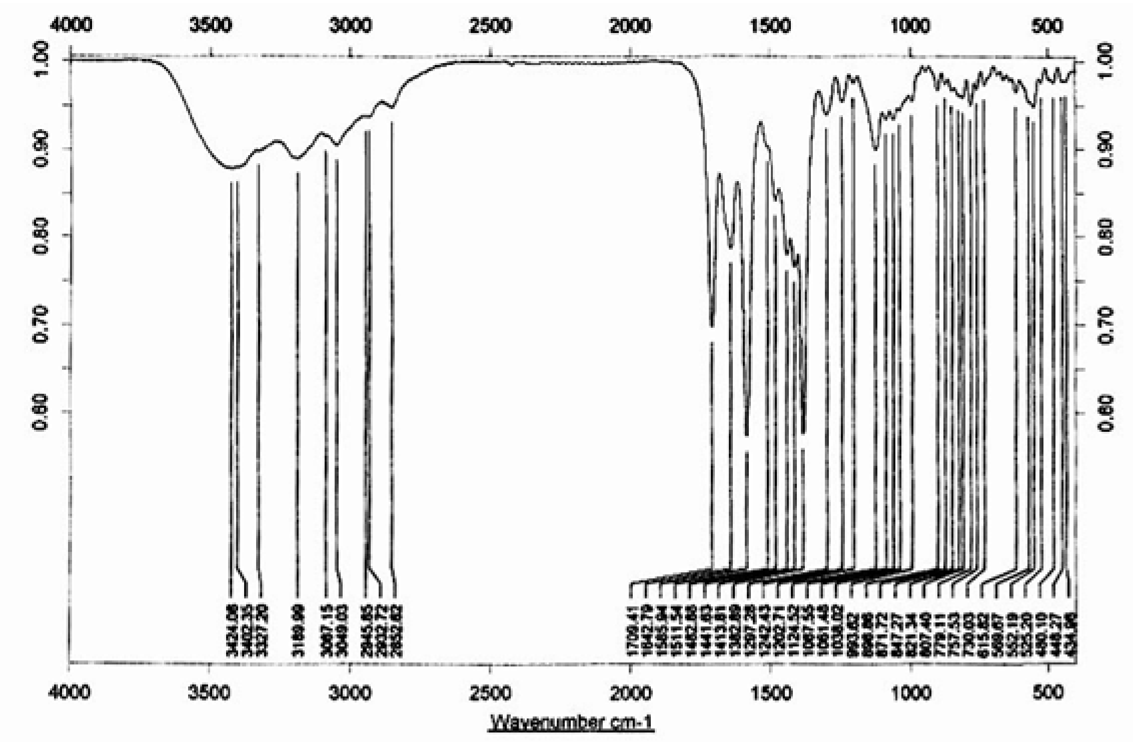

[Cu(HAcbHexim)(NO$_3$)]·1/2H$_2$O

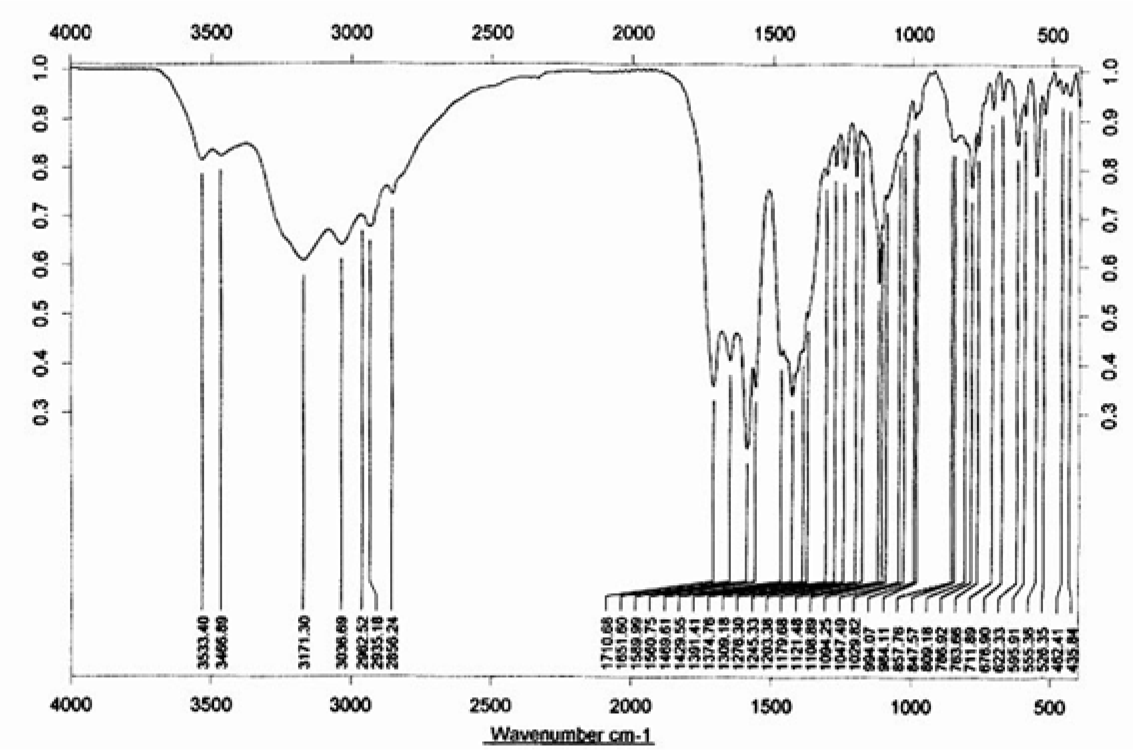

[Cu(HAcbHexim)ClO$_4$]

Figure S3. FT-IR spectra in the region 500-100 cm⁻¹

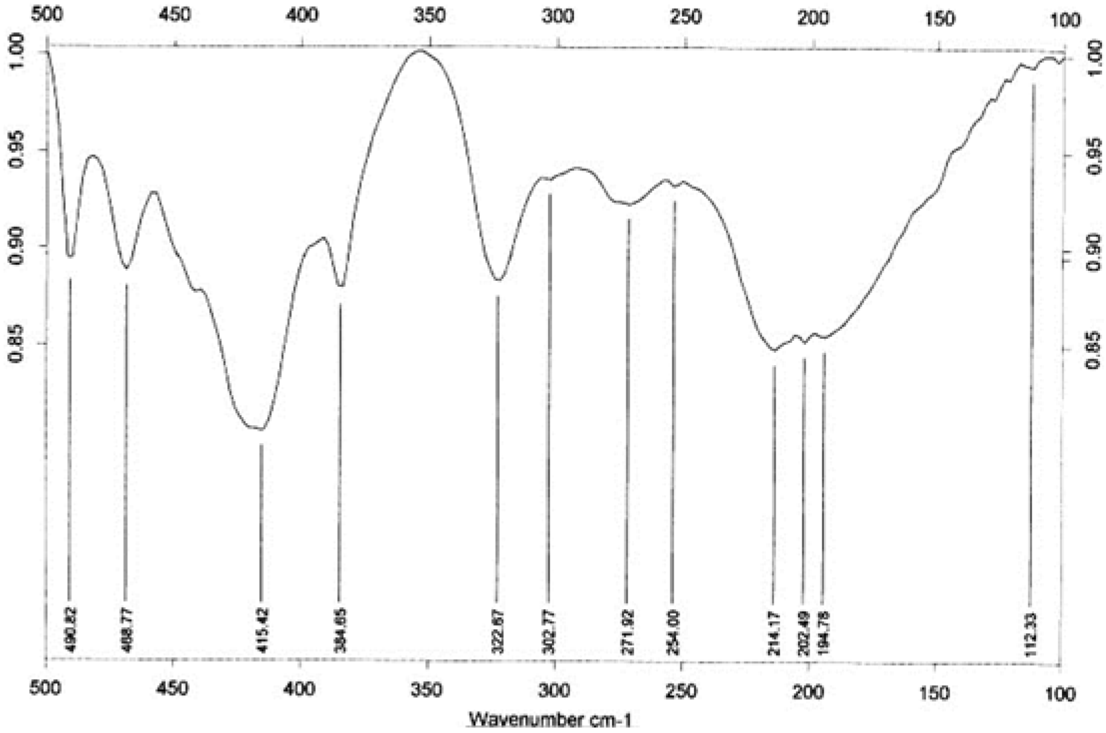

$H_2AcbDM\cdot H_2O$

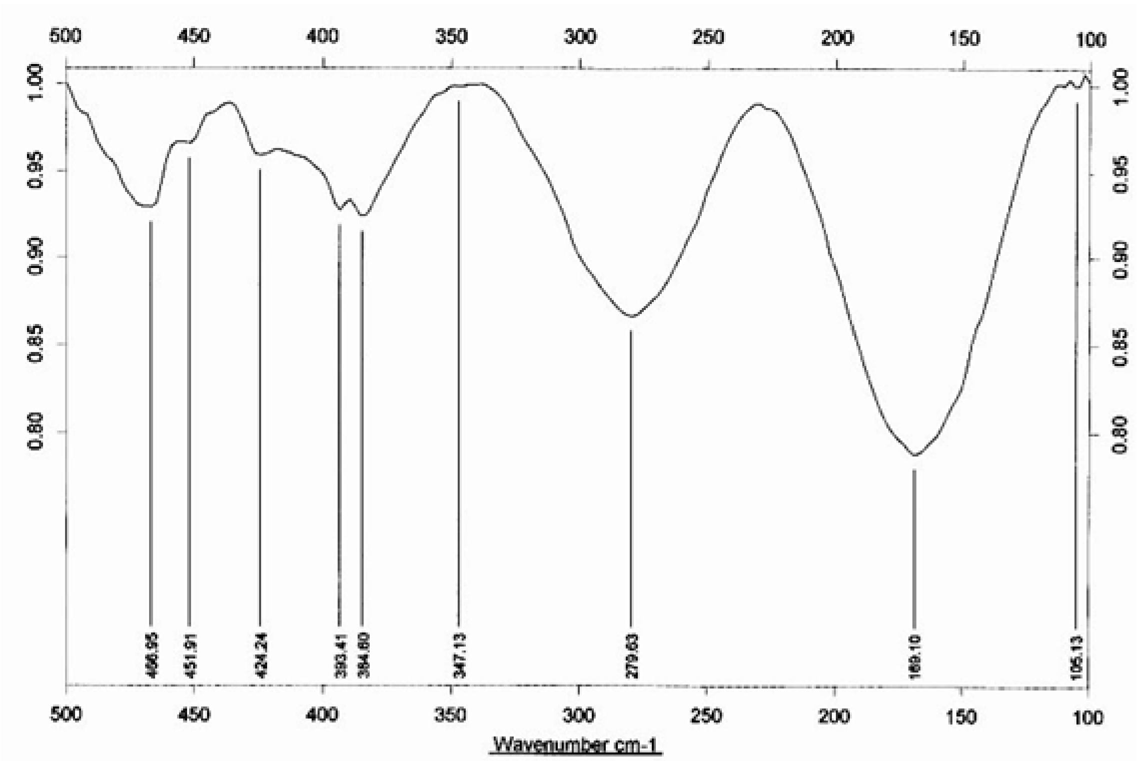

$H_2AcbHexim\cdot H_2O$

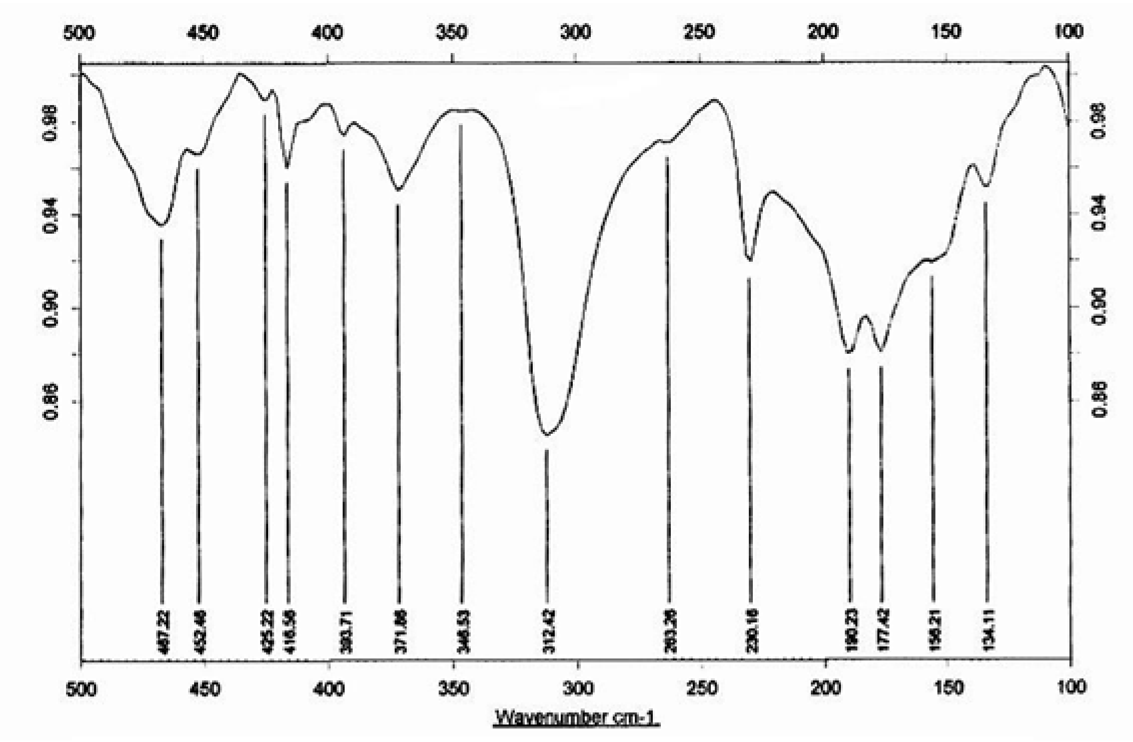

[Cu(HAcbDM)(OAc)]·3H₂O

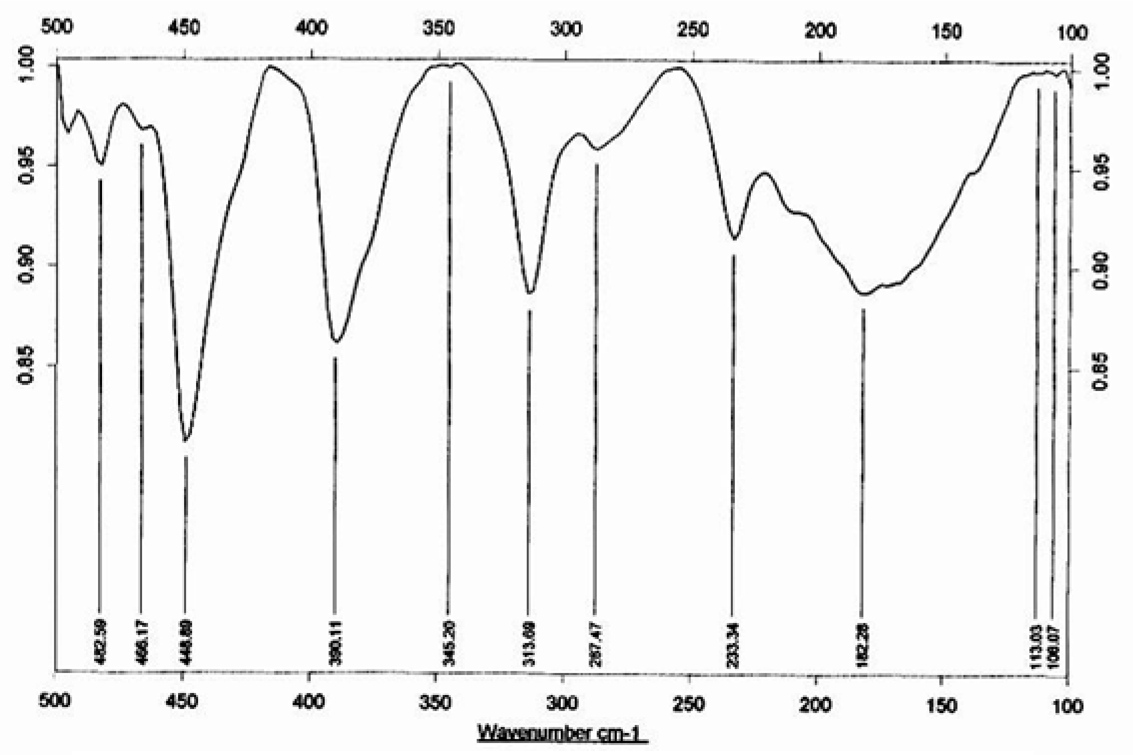

[Cu(HAcbDM)Cl]·1/2EtOH

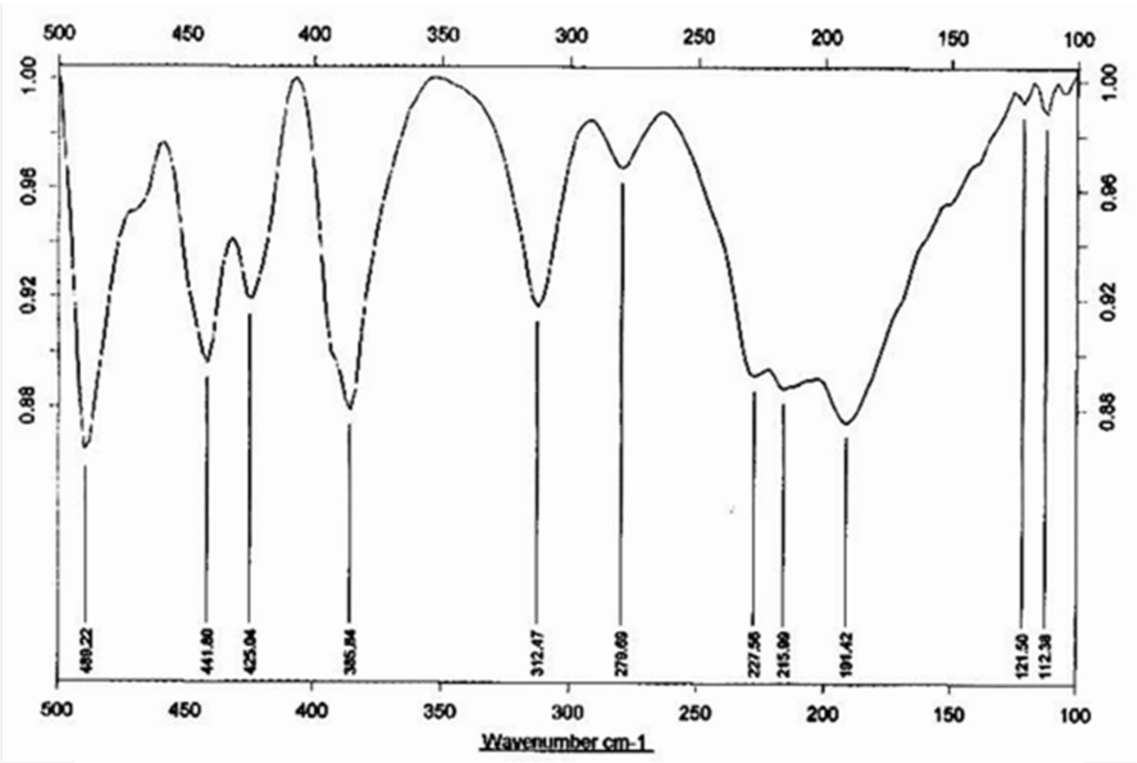

[Cu(HAcbDM)(NO₃)]

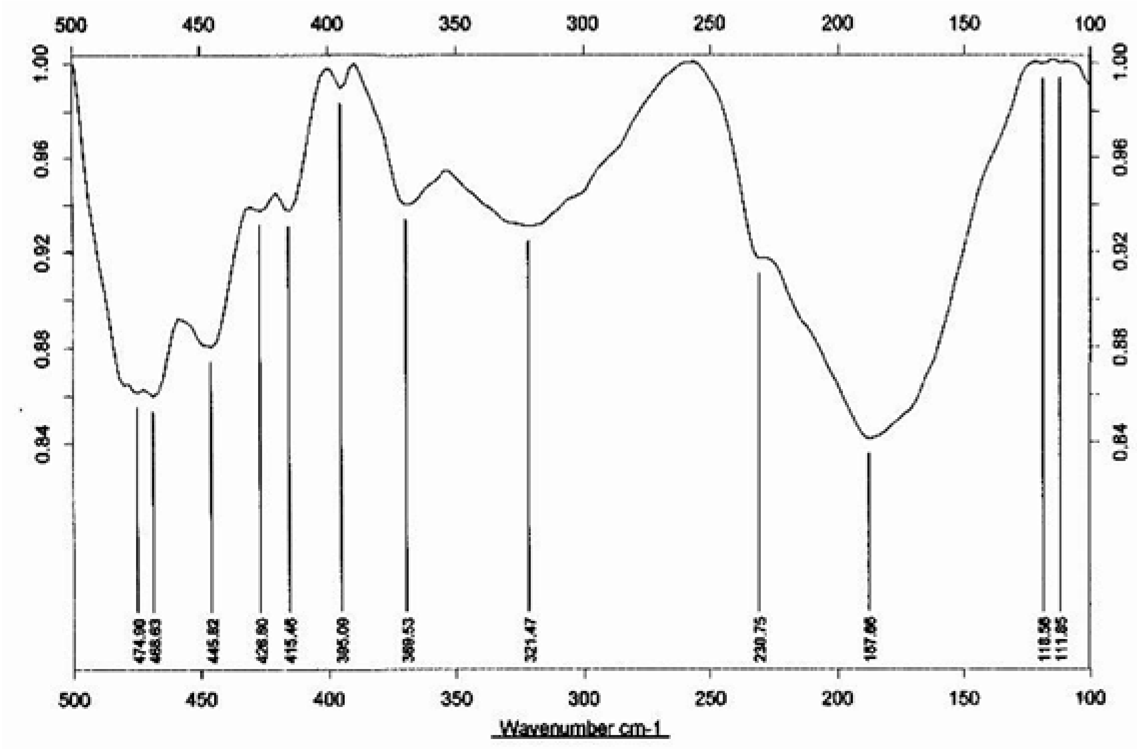

[Cu(HAcbDM)(H₂O)₂]ClO₄

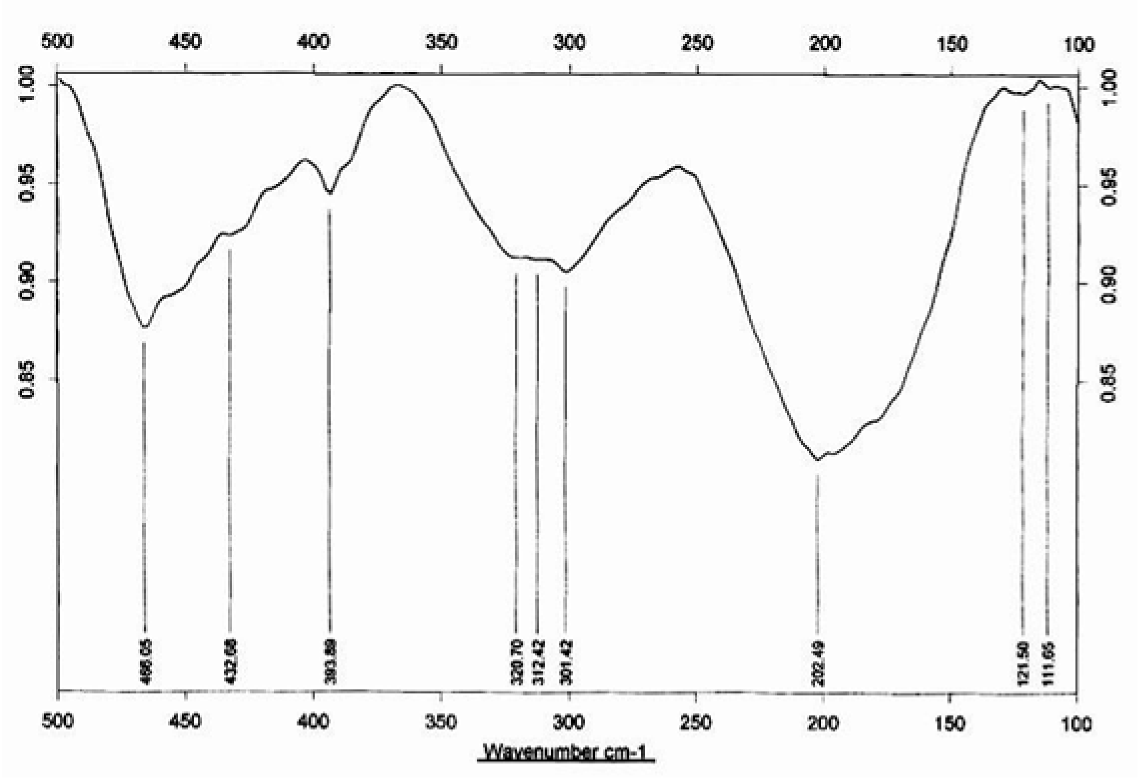

[Cu(HAcbHexim)(OAc)]·5H₂O

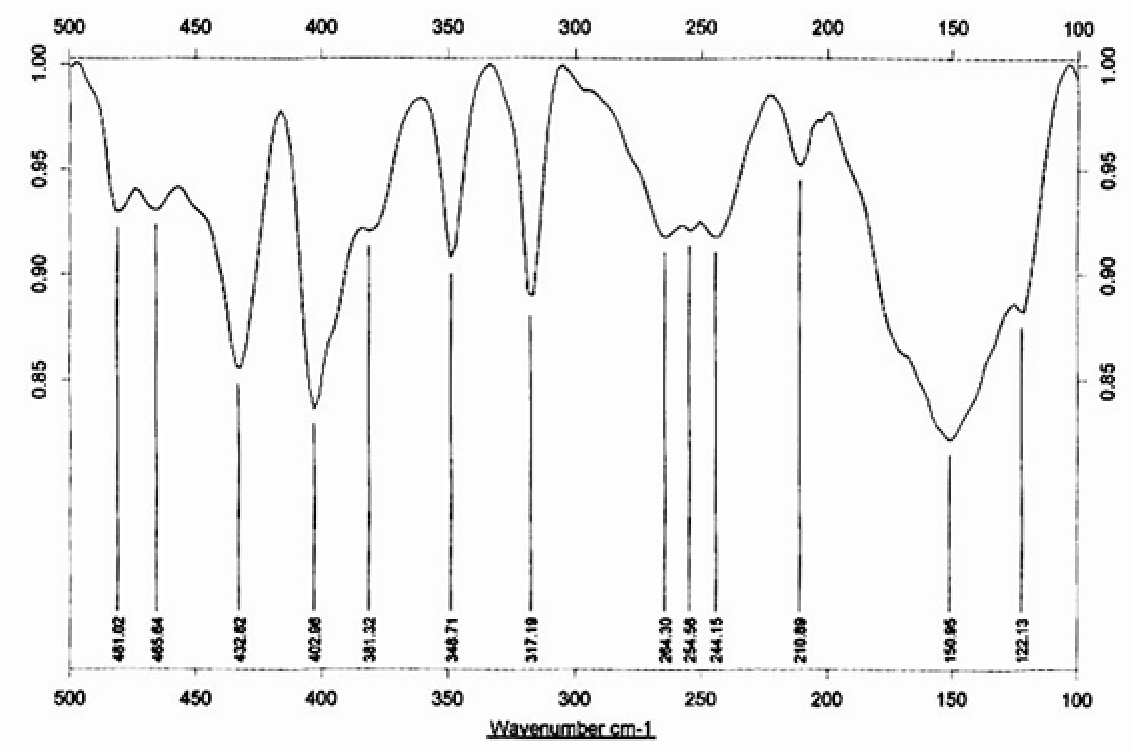

[Cu(HAcbHexim)Cl]·1/2EtOH

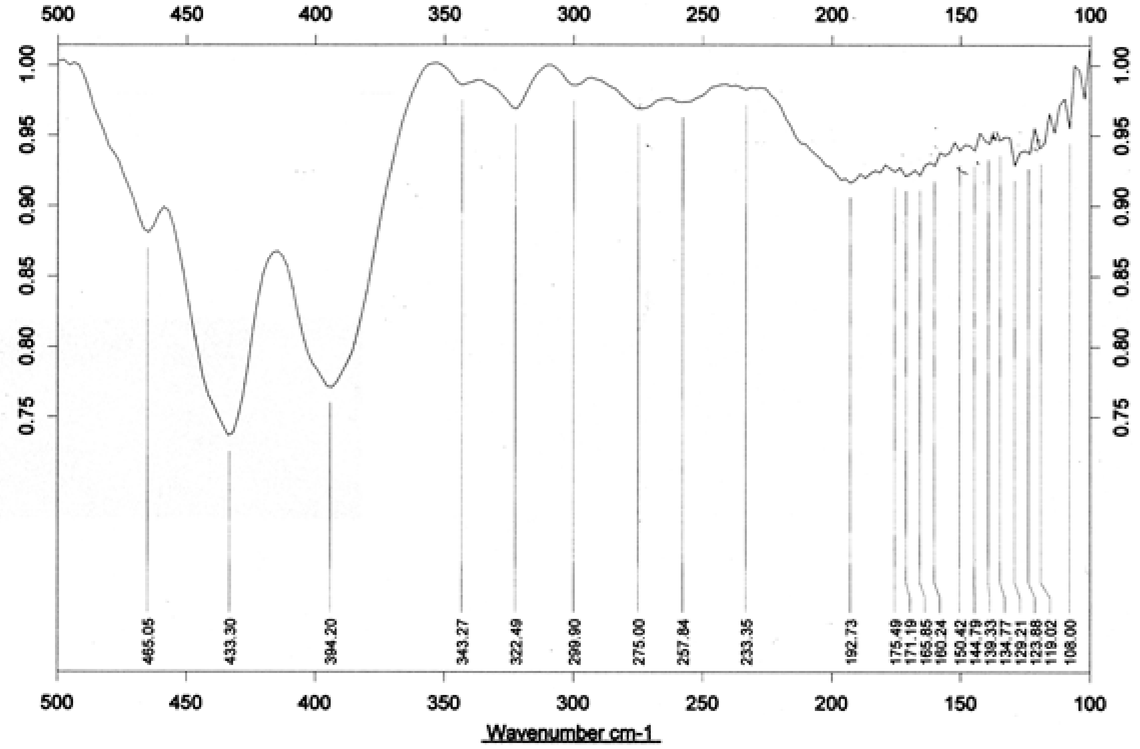

[Cu(HAcbHexim)(NO₃)]·1/2H₂O

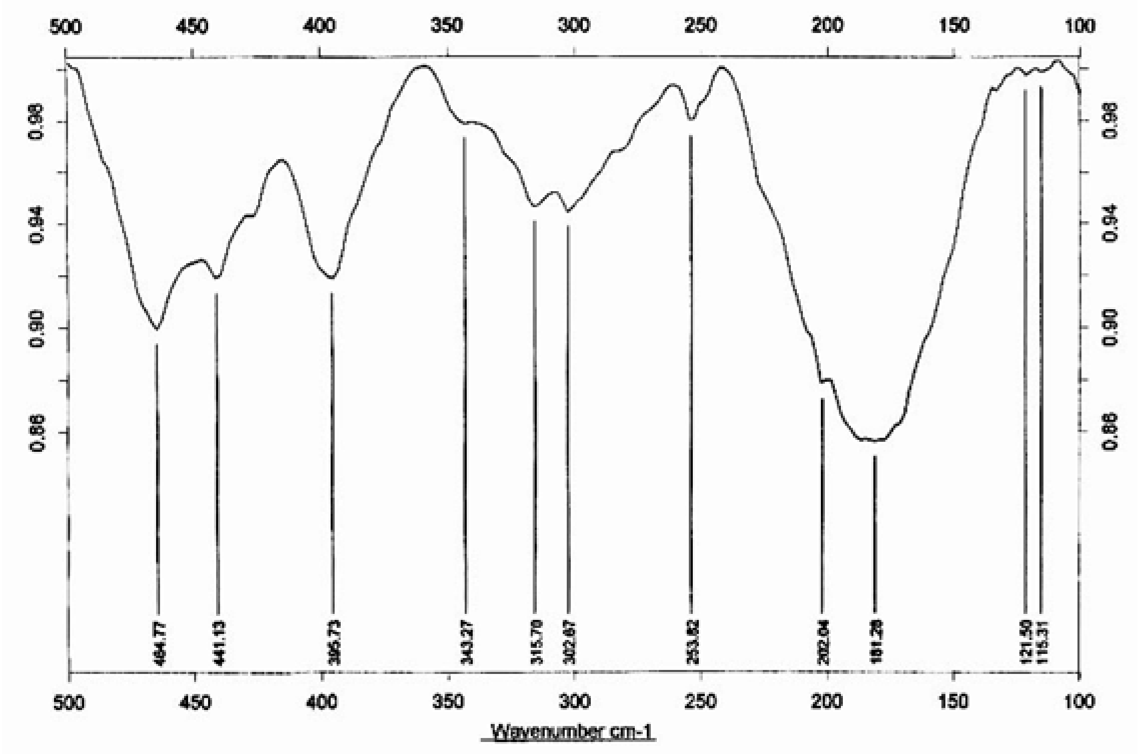

[Cu(HAcbHexim)ClO₄]

Figure S4. UV-visible Spectra

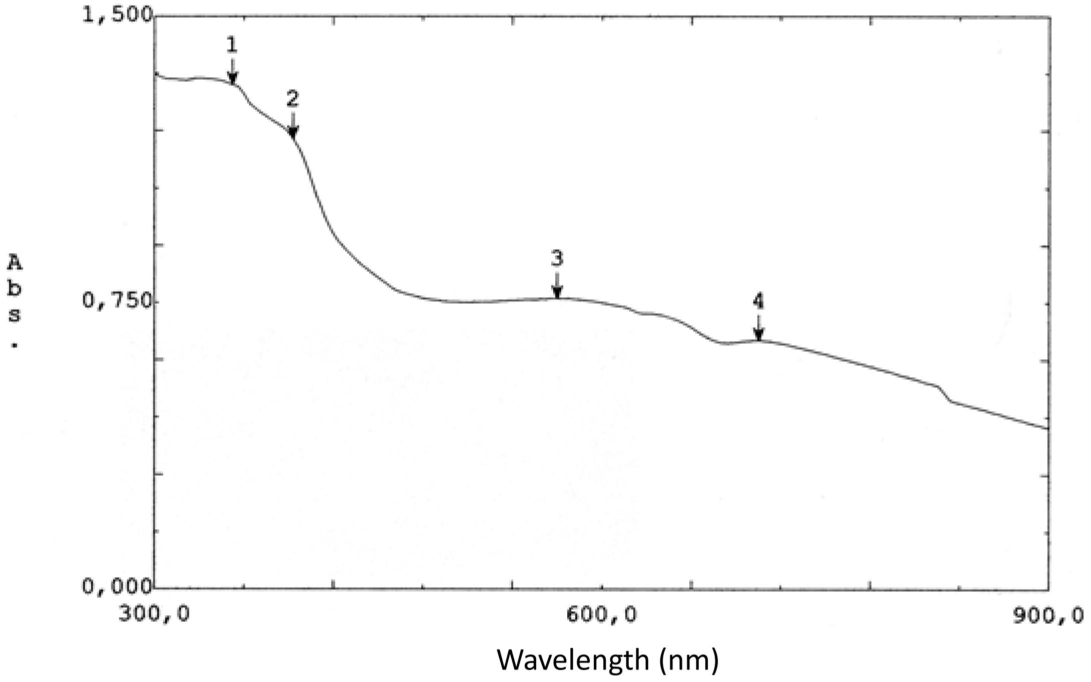

[Cu(HAcbDM)(OAc)]·3H₂O

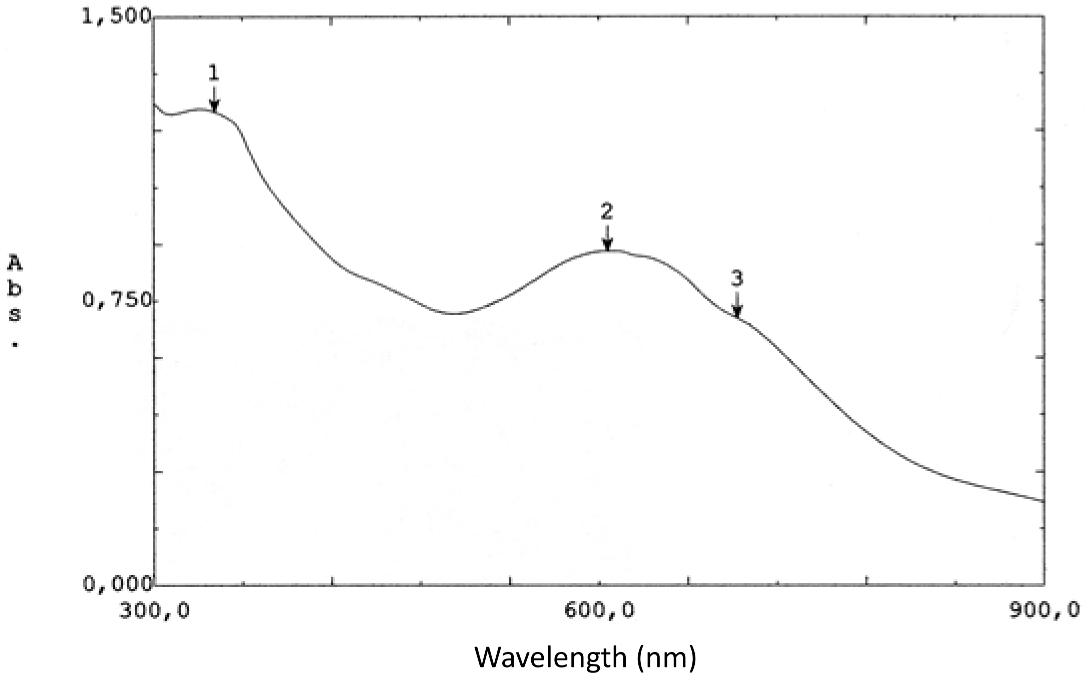

[Cu(HAcbDM)Cl]·1/2EtOH

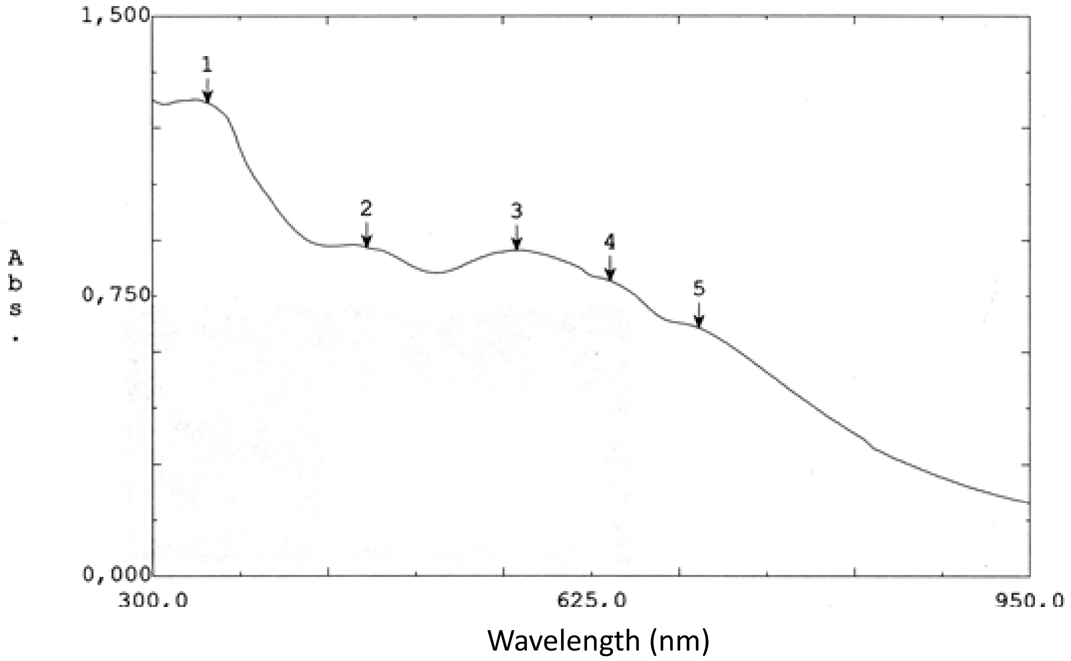

[Cu(HAcbDM)(NO$_3$)]

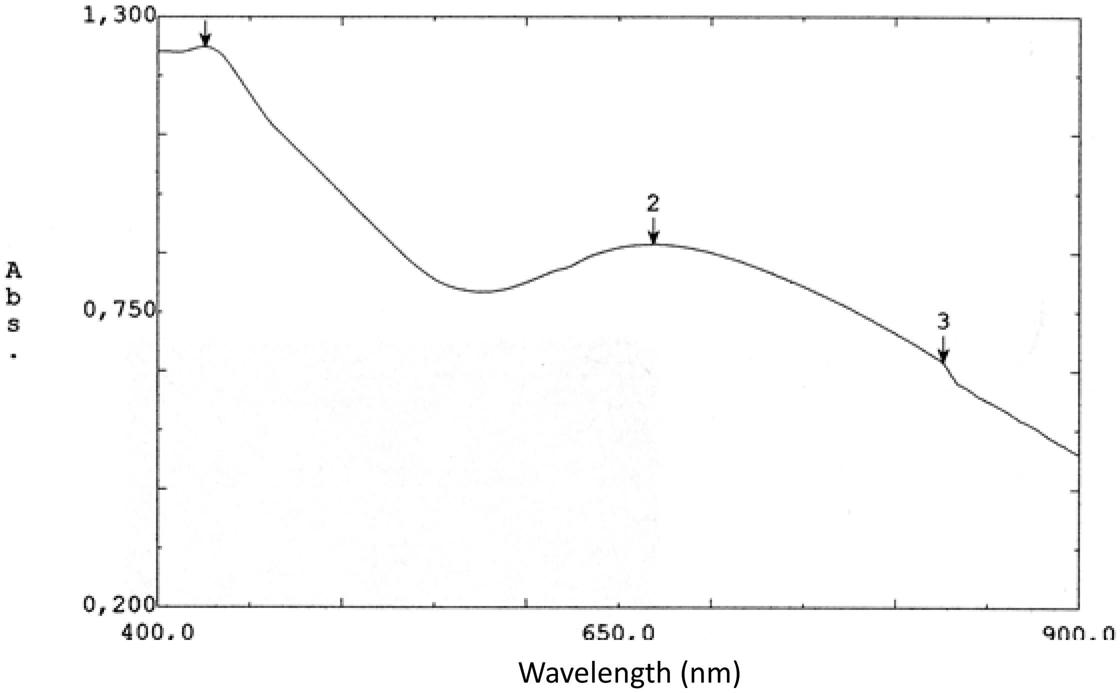

[Cu(HAcbDM)(H$_2$O)$_2$]ClO$_4$

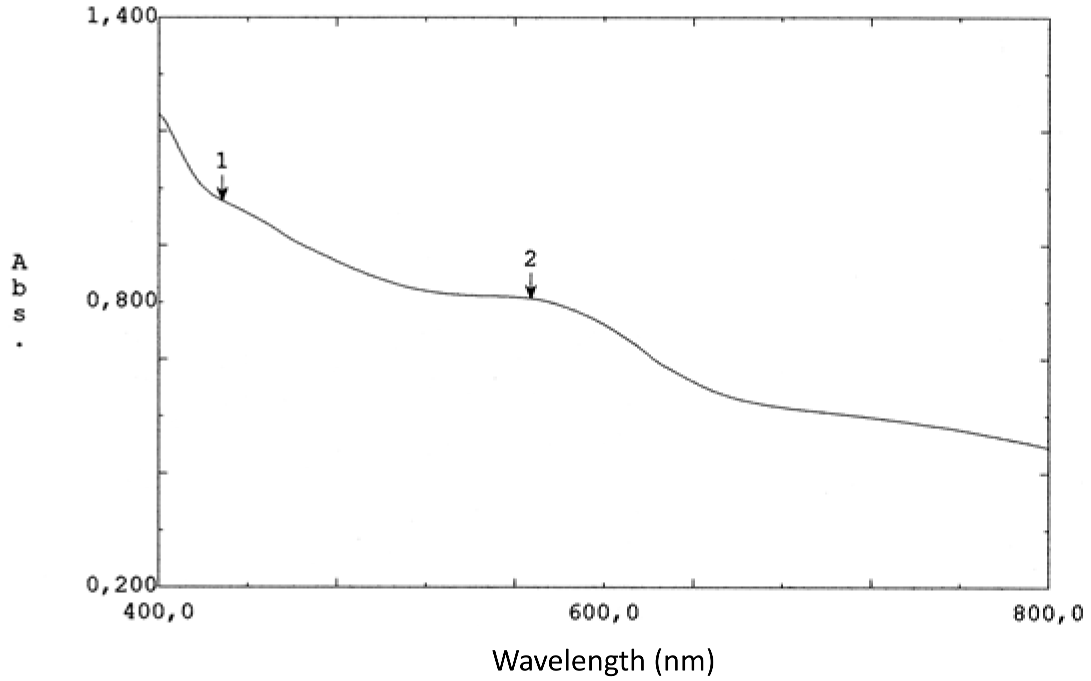

[Cu(HAcbHexim)(OAc)]·5H₂O

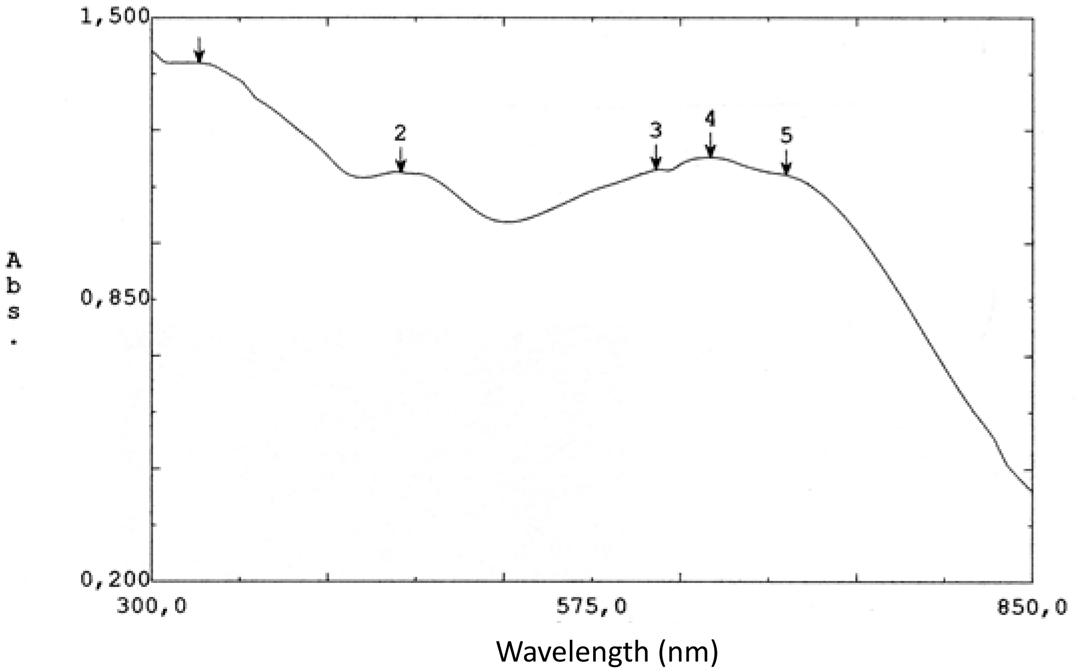

[Cu(HAcbHexim)Cl]·1/2EtOH

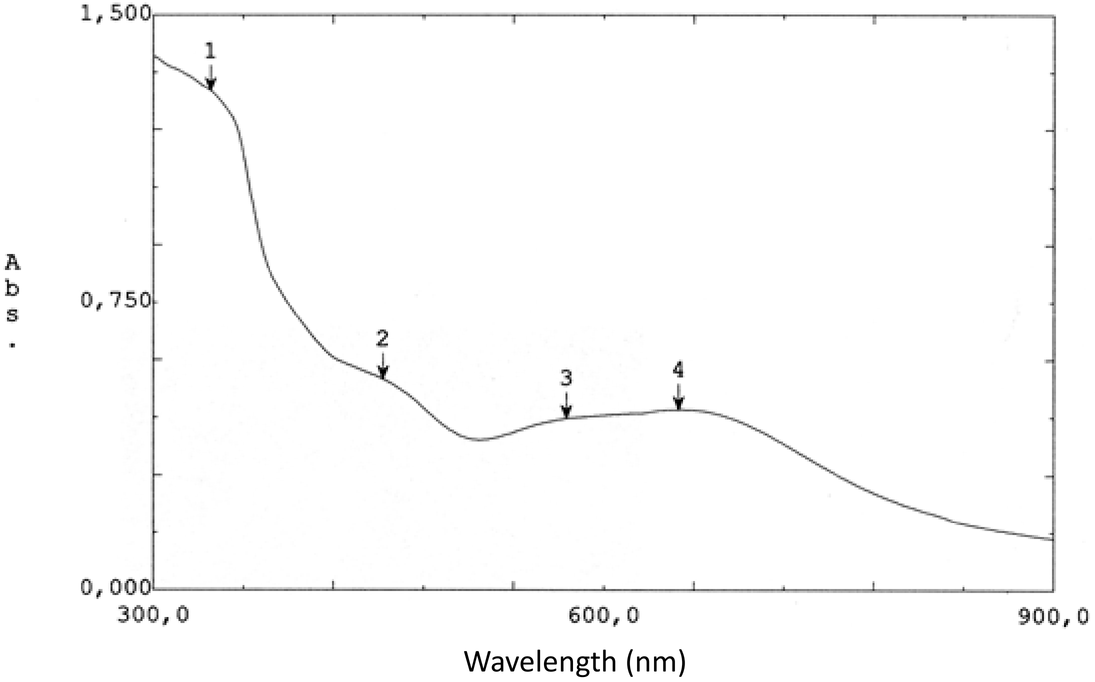

$[Cu(HAcbHexim)(NO_3)]\cdot 1/2H_2O$

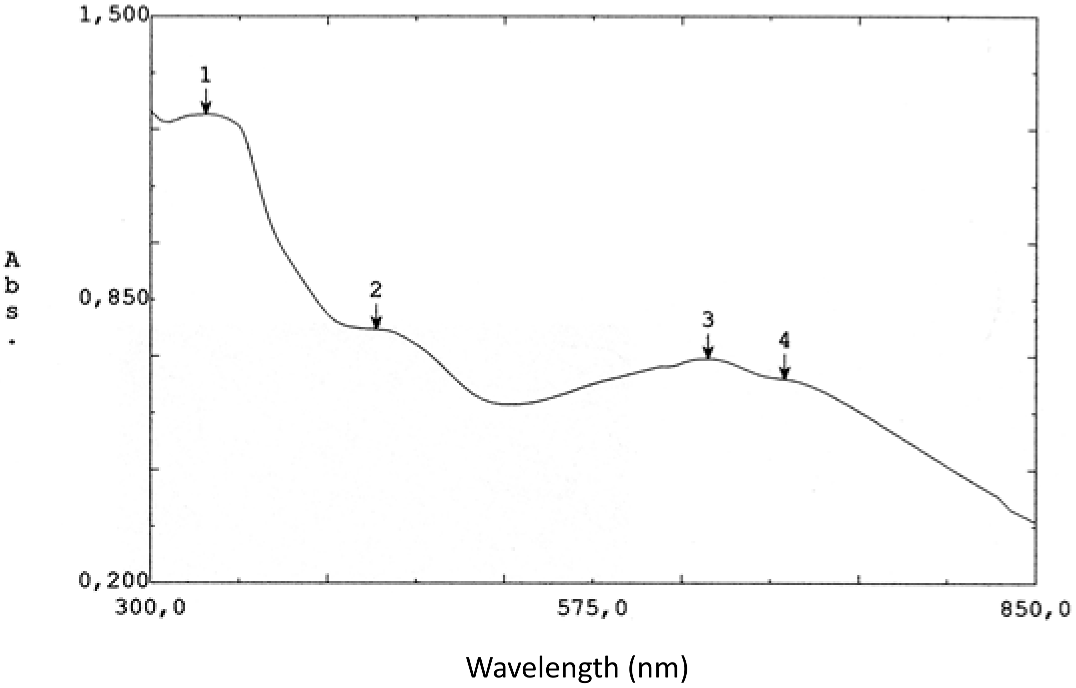

$[Cu(HAcbHexim)ClO_4]$

Figure S5. EPR spectra

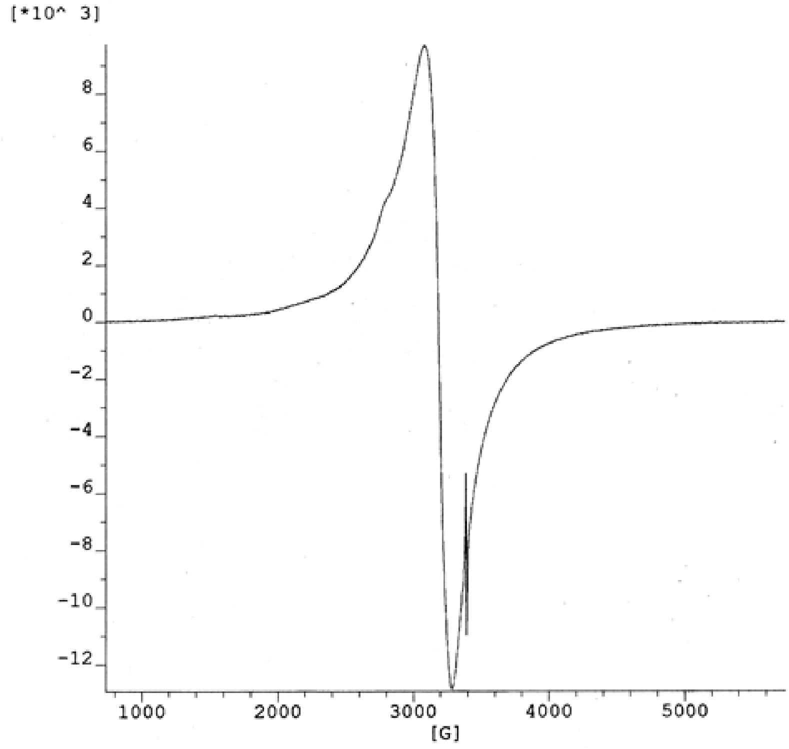

[Cu(HAcbDM)(OAc)]·3H2O

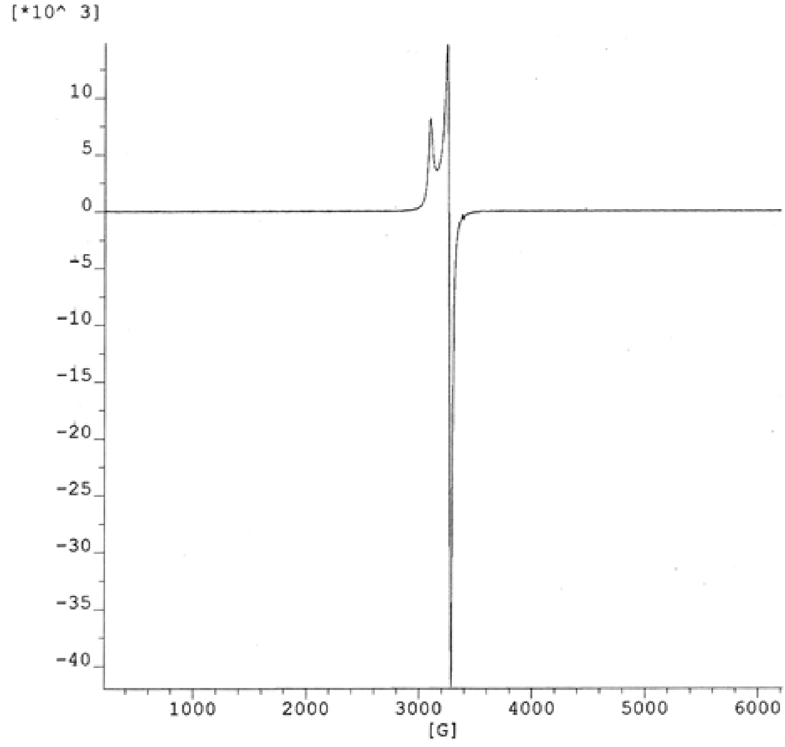

[Cu(HAcbDM)Cl]·1/2EtOH

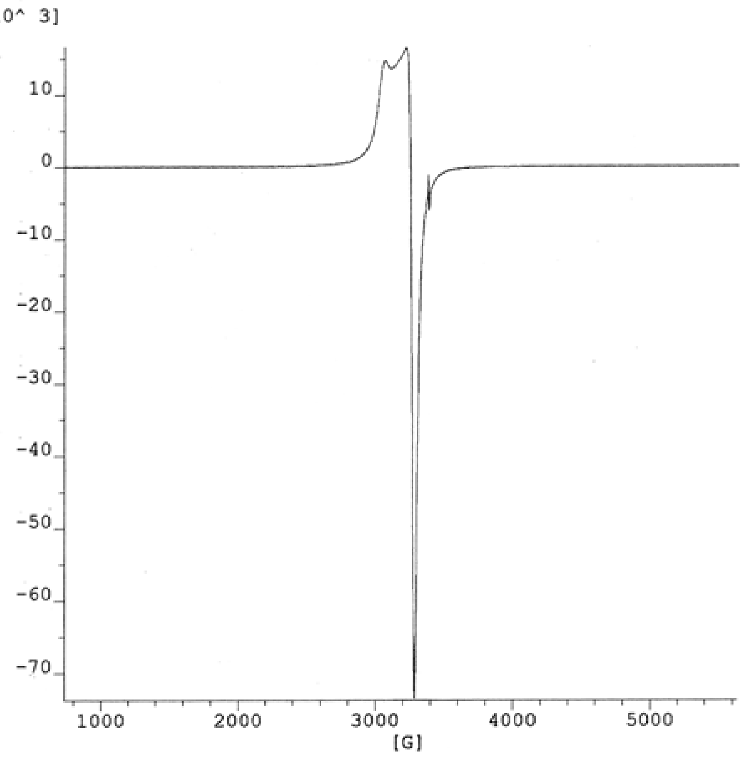

[Cu(HAcbDM)(NO$_3$)]

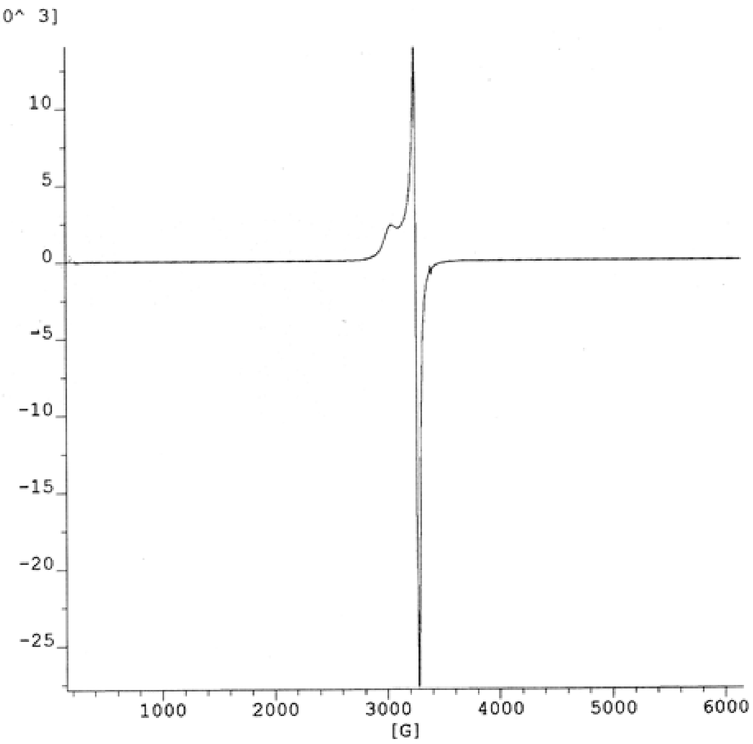

[Cu(HAcbDM)(H$_2$O)$_2$]ClO$_4$

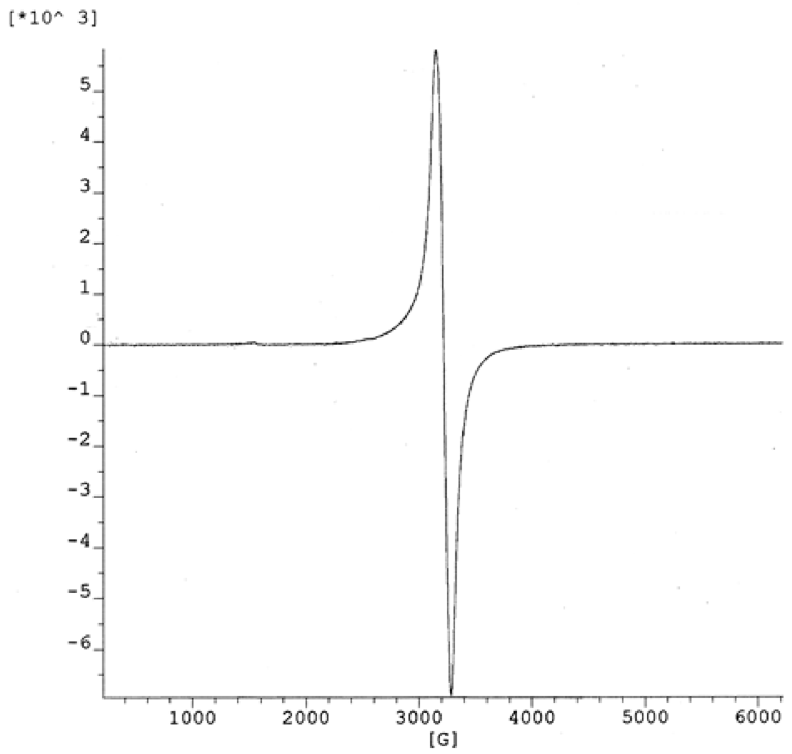

[Cu(HAcbHexim)(OAc)]·5H2O

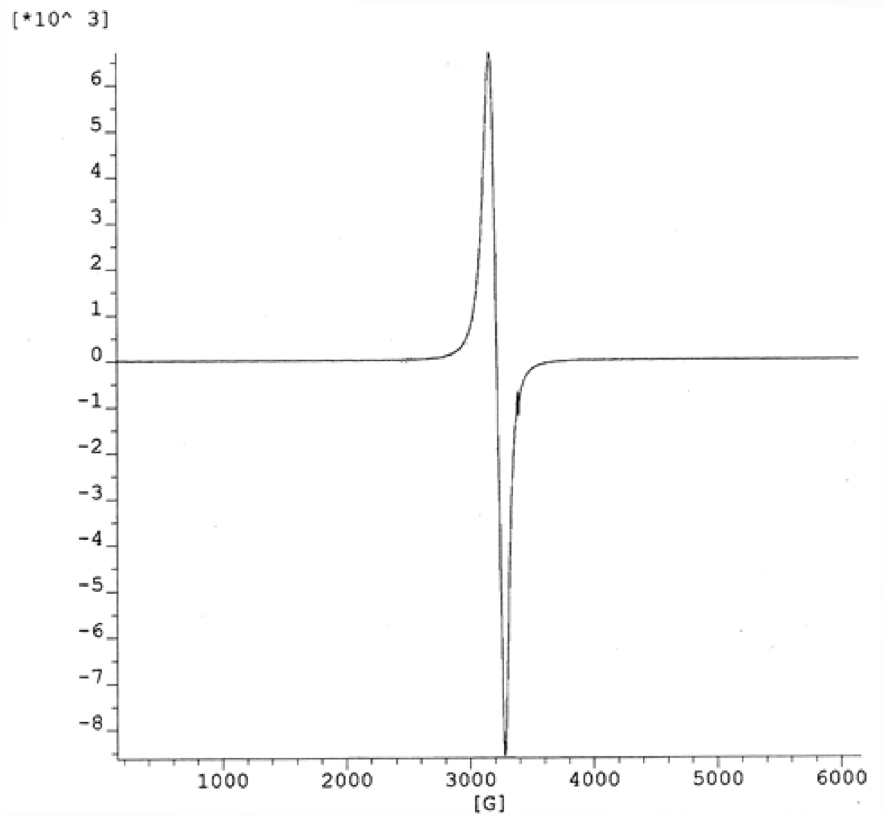

[Cu(HAcbHexim)Cl]·1/2EtOH

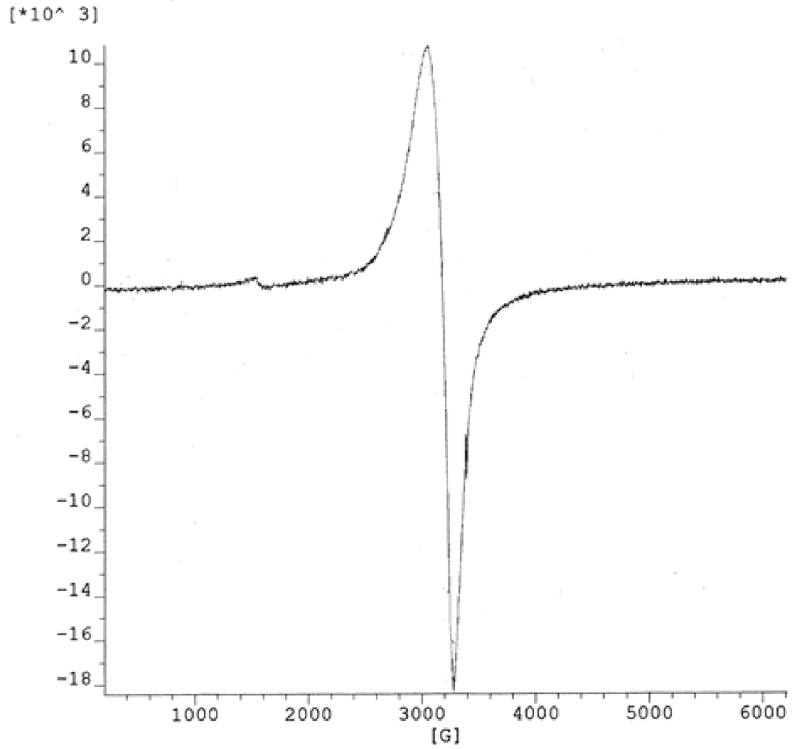

[Cu(HAcbHexim)(NO$_3$)]·1/2H$_2$O

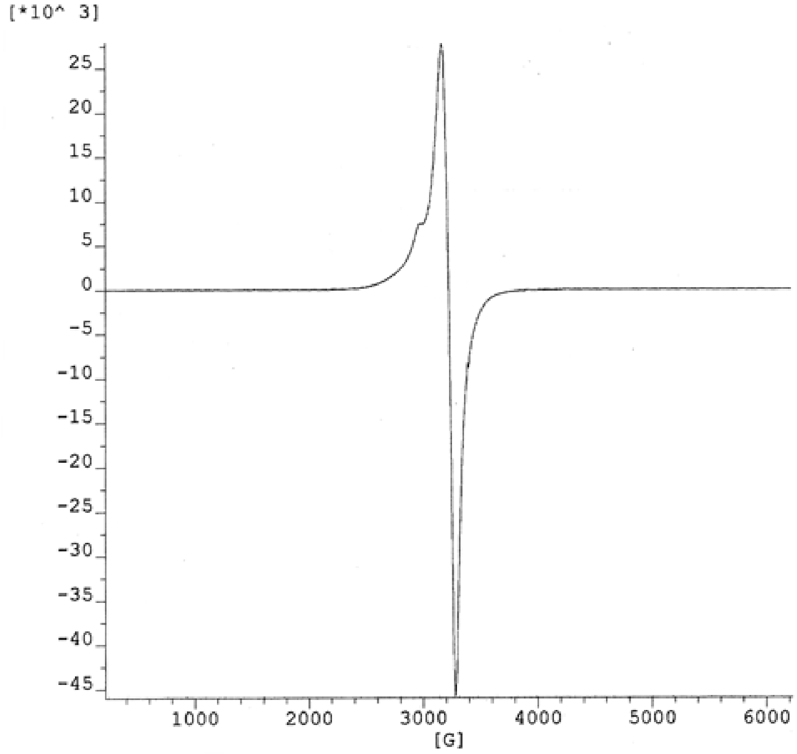

[Cu(HAcbHexim)ClO$_4$]

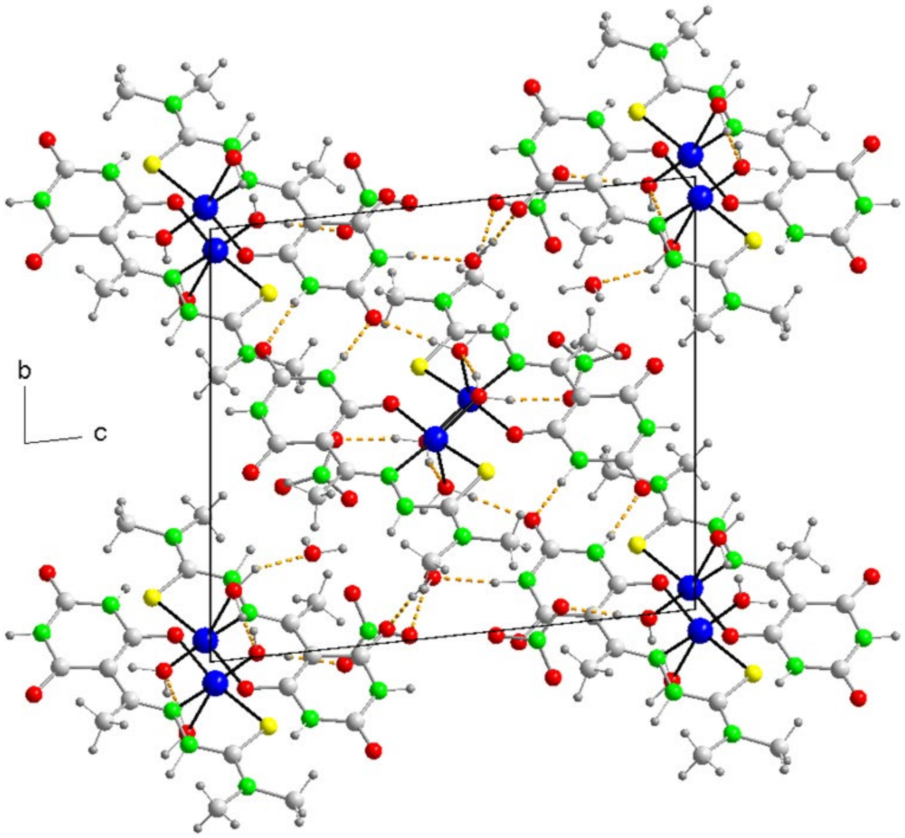

Figure S6. View of the unit cell for **3a** in the "bc" plane, showing the crystal packing. Hydrogen bonds are shows as orange dashed lines.

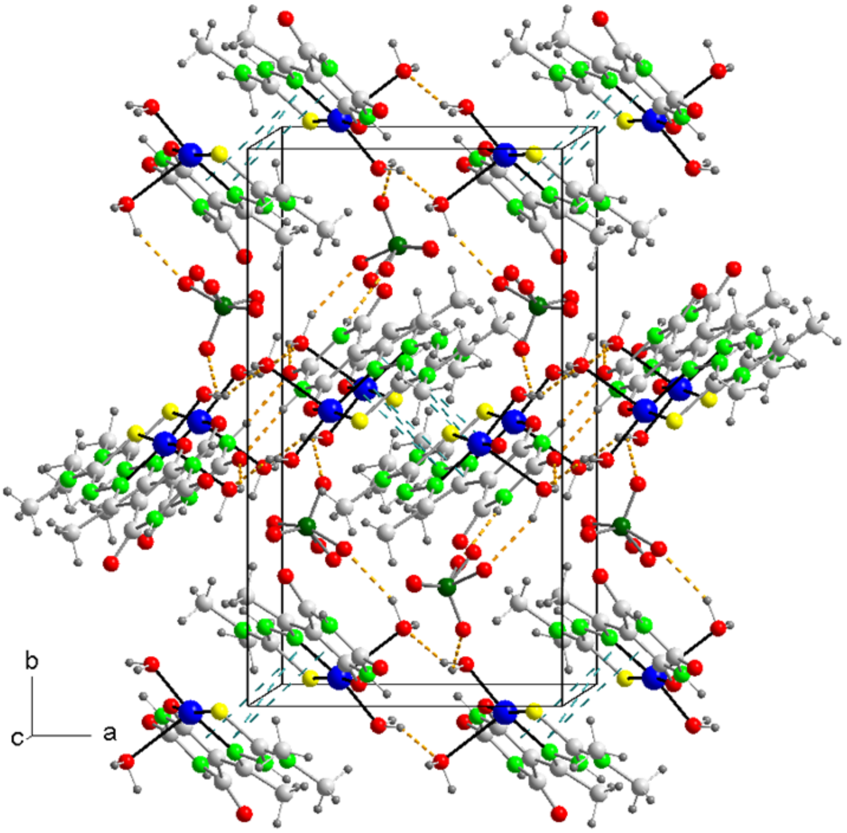

Figure S7. View of the unit cell for **4** in the "bc" plane, showing the crystal packing, the hydrogen bonding (orange dashed lines) and π-π stacking interactions (blue dashed lines).

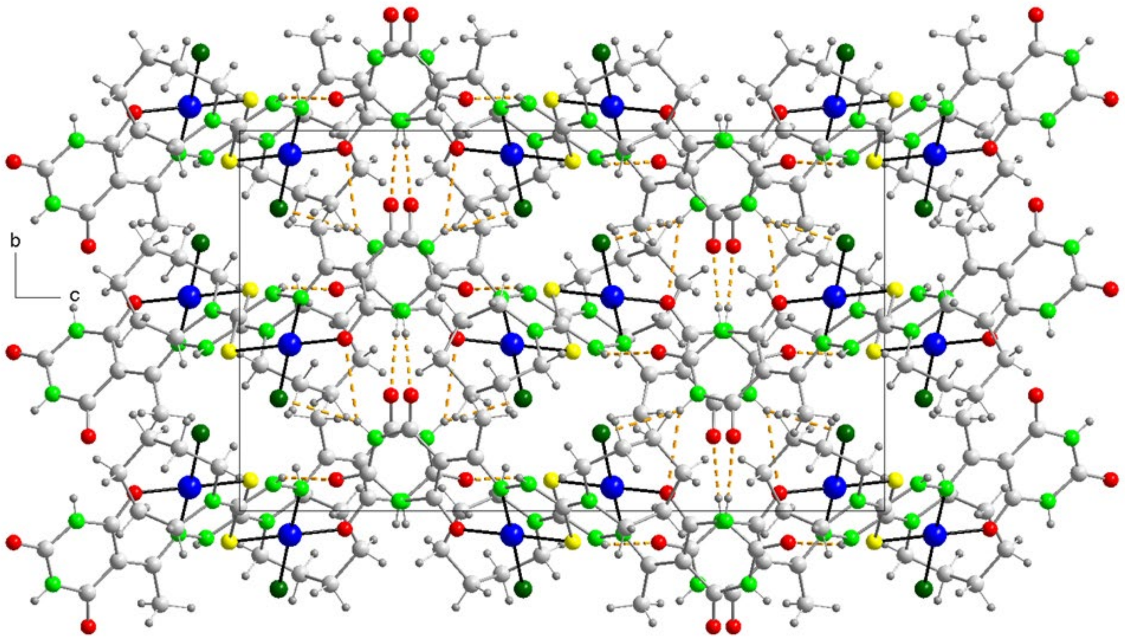

Figure S8. View of the unit cell for **6** in the "bc" plane, showing the crystal packing. Hydrogen bonds are shows as orange dashed lines.

Figure S9. Cyclic voltammograms

[Cu(HAcbDM)(OAc)]·3H₂O

**$E_{initial}$:**	0.900 V	**E_{vertex}:**	-2.199 V	**Speed:**	0.020 V/s
**E_{final}:**	0.900 V	**Delay:**	10 s		

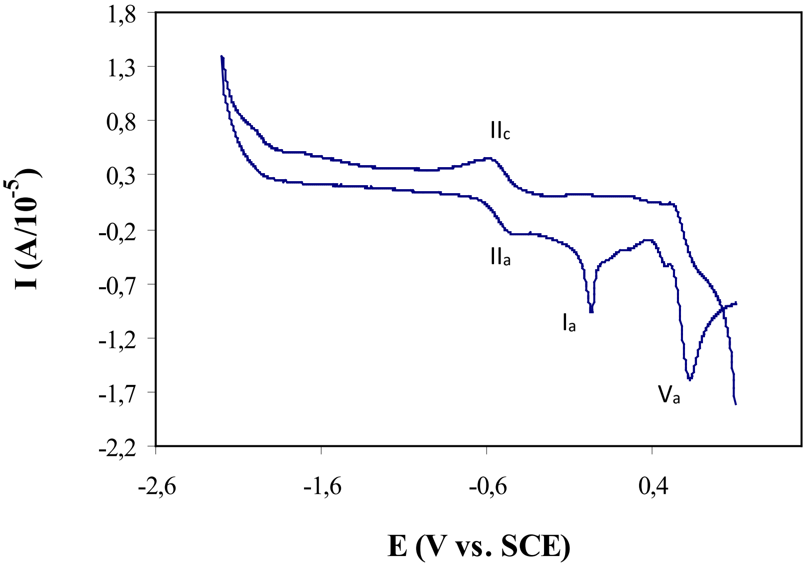

Peak Potentials

System	$(E_p)_c$	$(E_p)_a$	$(E_p)_a - (E_p)_c$	$[(E_p)_a + (E_p)_c]/2$	$(E_{p/2})_c$	$(E_{p/2})_c - (E_p)_c$	$(E_{1/2})_c$	E°'
**I:**		0.033						
**II:**	-0.582	-0.396	0.186	-0.489	-0.471	0.111	-0.499	-0.553
**V:**		0.624						

[Cu(HAcbDM)(OAc)]·3H₂O (**1**·3H₂O)

**$E_{initial}$:**	0.400 V	**E_{vertex}:**	-1.200 V	**Speed:**	0.200 V/s
**E_{final}:**	0.400 V	**Delay:**	0 s		

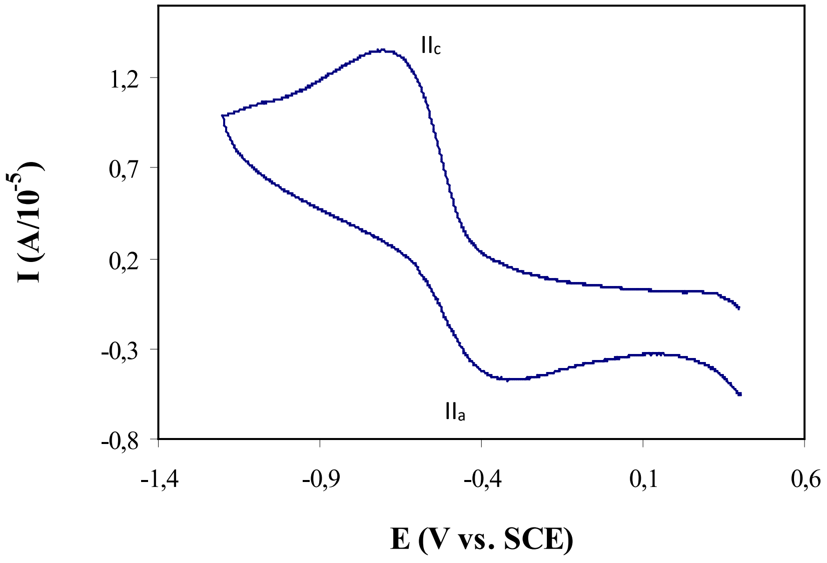

Peak Potentials

System	$(E_p)_c$	$(E_p)_a$	$(E_p)_a - (E_p)_c$	$[(E_p)_a + (E_p)_c]/2$	$(E_{p/2})_c$	$(E_{p/2})_c - (E_p)_c$	$(E_{1/2})_c$	$E^{o'}$
**II:**	-0.708	-0.371	0.337	-0.539	-0.518	0.190	-0.545	-0.679

[Cu(HAcbDM)(OAc)]·3H₂O (**1**·3H₂O)

**$E_{initial}$:**	0.900 V	**E_{vertex}:**	-1.098 V	**Speed:**	0.200 V/s
**E_{final}:**	0.900 V	**Delay:**	10 s		

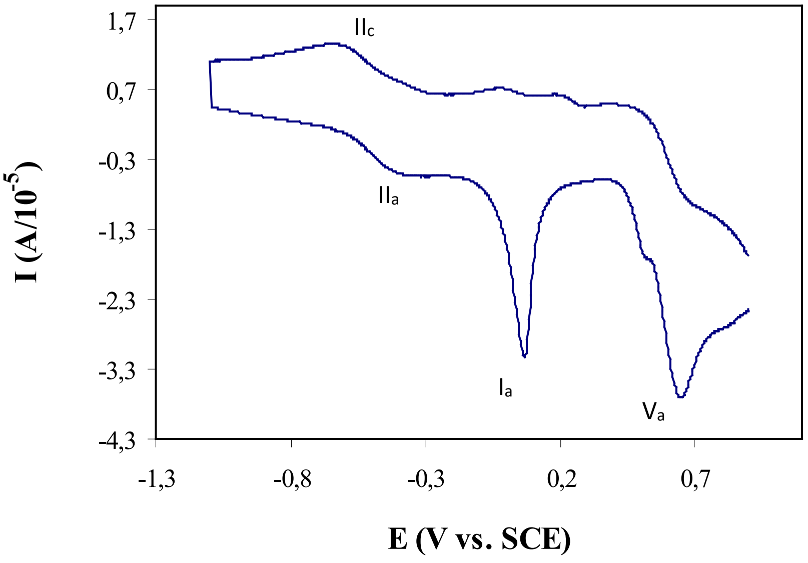

(No peak potentials have been included)

[Cu(HAcbDM)(OAc)]·3H₂O (**1**·3H₂O)

**$E_{initial}$:**	0.900 V	**E_{vertex}:**	-0.999 V	**Speed:**	0.200 V/s
**E_{final}:**	0.900 V	**Delay:**	10 s		

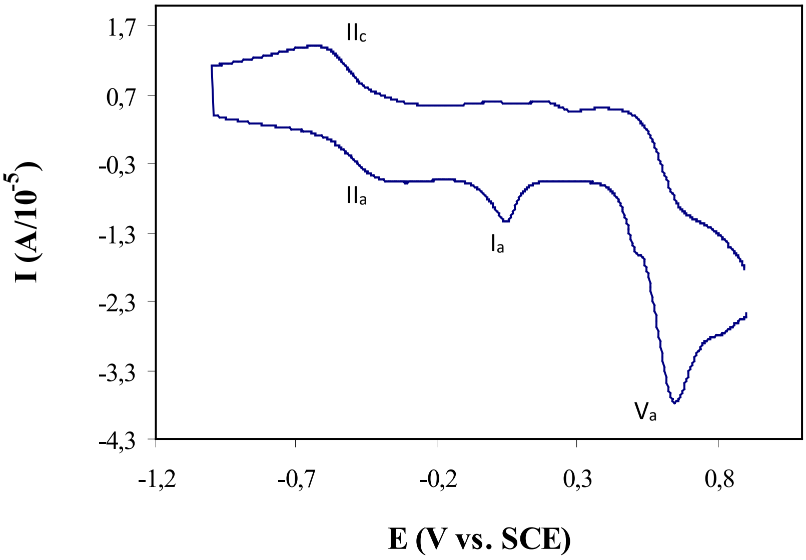

(No peak potentials have been included)

[Cu(HAcbDM)(OAc)]·3H₂O (**1**·3H₂O)

**$E_{initial}$:**	0.900 V	**E_{vertex}:**	-0.300 V	**Speed:**	0.200 V/s
**E_{final}:**	0.900 V	**Delay:**	0 s		

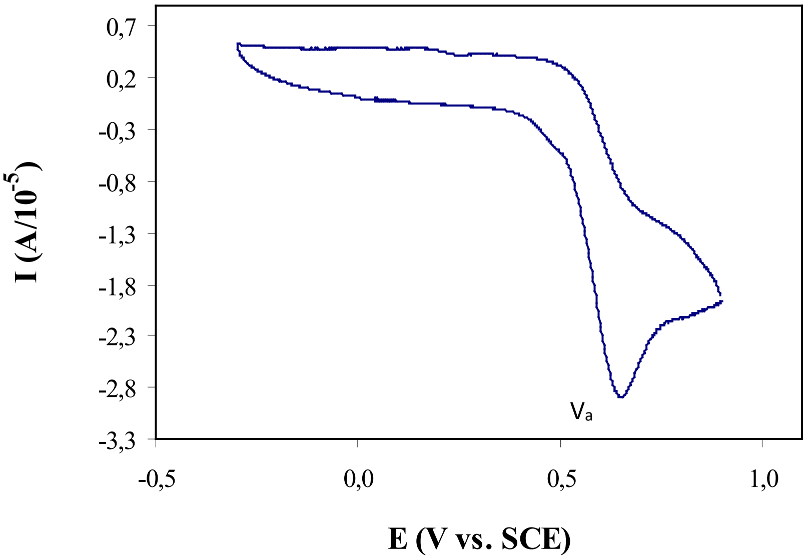

Peak Potentials

System	$(E_p)_c$	$(E_p)_a$	$(E_p)_a - (E_p)_c$	$[(E_p)_a + (E_p)_c]/2$	$(E_{p/2})_c$	$(E_{p/2})_c - (E_p)_c$	$(E_{1/2})_c$	$E^{o\prime}$
**V:**		0.648						

[Cu(HAcbDM)Cl]·1/2EtOH (**2**·1/2EtOH)

**$E_{initial}$:**	0.900 V	**E_{vertex}:**	-2.000 V	**Speed:**	0.200 V/s
**E_{final}:**	0.900 V	**Delay:**	0 s		

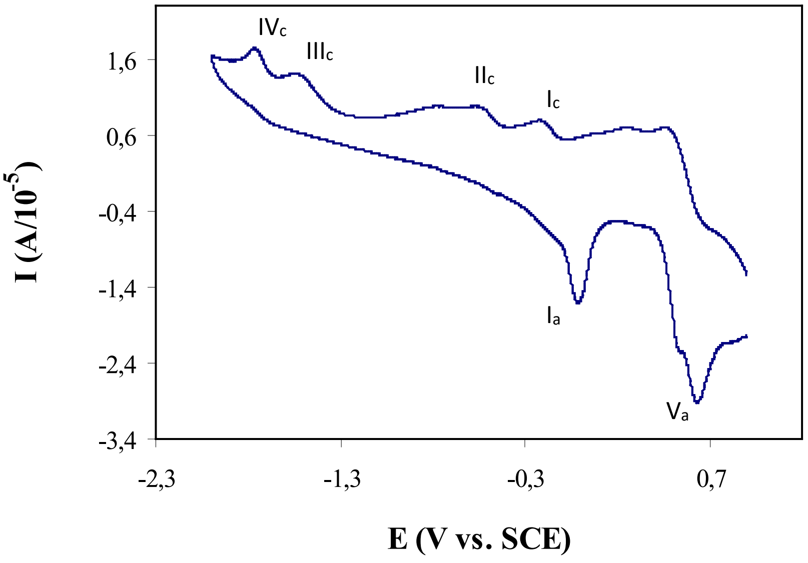

Peak Potentials

System	$(E_p)_c$	$(E_p)_a$	$(E_p)_a$ - $(E_p)_c$	$[(E_p)_a + (E_p)_c]/2$	$(E_{p/2})_c$	$(E_{p/2})_c$ - $(E_p)_c$	$(E_{1/2})_c$	$E^{o\prime}$
**I:**	-0.216	-0.015	0.201	-0.115	-0.156	0.060	-0.184	-0.188
**II:**	-0.558				-0.482	0.076	-0.510	-0.530
**III:**	-1.534				-1.428	0.106	-1.456	-1.506
**IV:**	-1.766				-1.714	0.052	-1.741	-1.737
**V:**		0.631						

[Cu(HAcbDM)Cl]·1/2EtOH (**2**·1/2EtOH)

$E_{initial}$: 0.400 V **E_{vertex}:** -0.800 V **Speed:** 0.200 V/s
E_{final}: 0.400 V **Delay:** 0 s

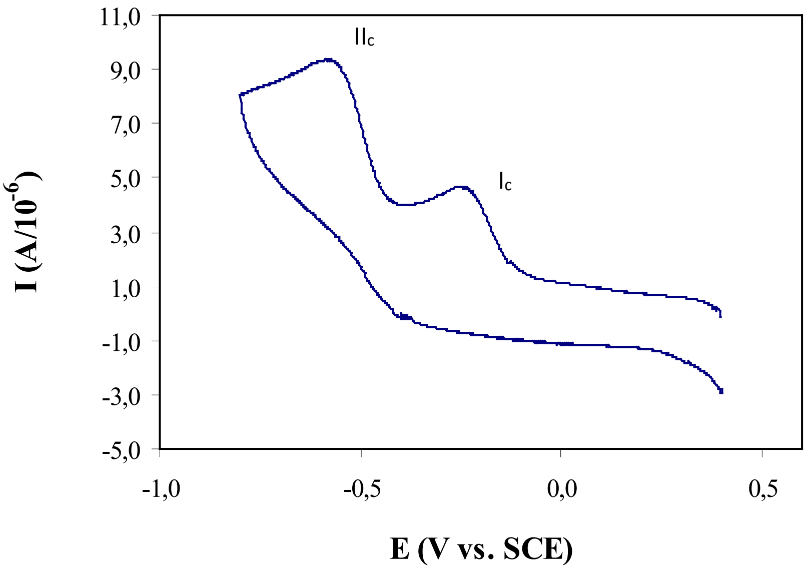

Peak Potentials

System	$(E_p)_c$	$(E_p)_a$	$(E_p)_a - (E_p)_c$	$[(E_p)_a + (E_p)_c]/2$	$(E_{p/2})_c$	$(E_{p/2})_c - (E_p)_c$	$(E_{1/2})_c$	$E^{o\prime}$
**I:**	-0.234				-0.170	0.064	-0.197	-0.205
**II:**	-0.582				-0.494	0.088	-0.521	-0.553

[Cu(HAcbDM)Cl]·1/2EtOH (**2**·1/2EtOH)

E$_{initial}$: 0.400 V **E$_{vertex}$:** -0.800 V **Speed:** 0.200 V/s

E$_{final}$: 0.400 V **Delay:** 10 s

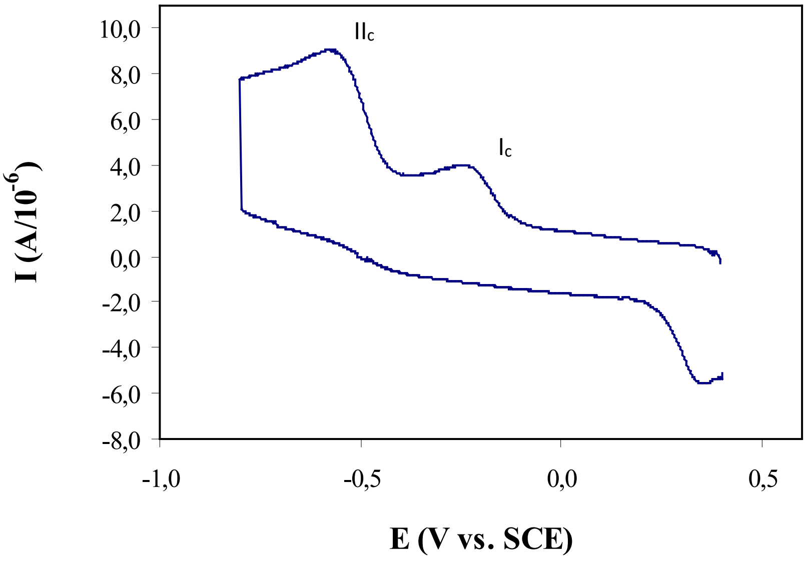

Peak Potentials

System	$(E_p)_c$	$(E_p)_a$	$(E_p)_a - (E_p)_c$	$[(E_p)_a + (E_p)_c]/2$	$(E_{p/2})_c$	$(E_{p/2})_c - (E_p)_c$	$(E_{1/2})_c$	$E^{o\prime}$
**I:**	-0.238				-0.168	0.070	-0.195	-0.209
**II:**	-0.576				-0.490	0.086	-0.517	-0.547

[Cu(HAcbDM)Cl]·1/2EtOH (**2**·1/2EtOH)

**$E_{initial}$:**	0.400 V	**E_{vertex}:**	-1.300 V	**Speed:**	0.200 V/s
**E_{final}:**	0.400 V	**Delay:**	10 s		

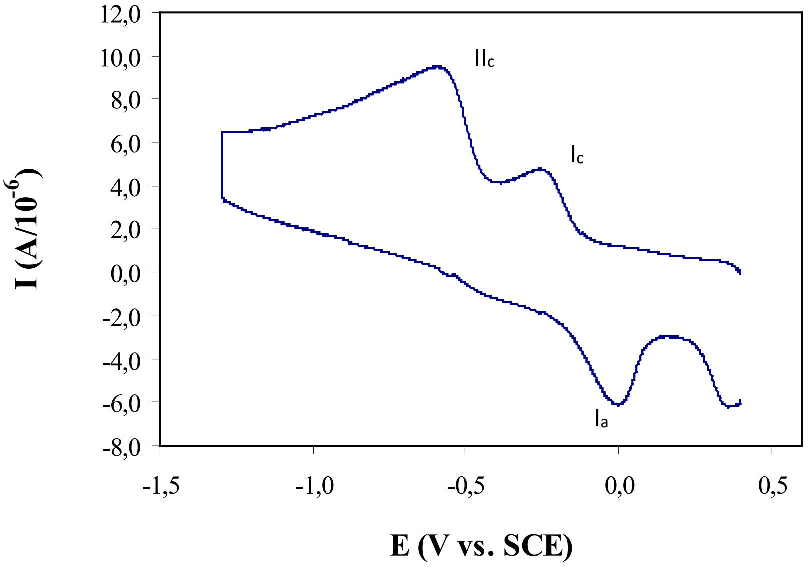

Peak Potentials

System	$(E_p)_c$	$(E_p)_a$	$(E_p)_a - (E_p)_c$	$[(E_p)_a + (E_p)_c]/2$	$(E_{p/2})_c$	$(E_{p/2})_c - (E_p)_c$	$(E_{1/2})_c$	$E^{o'}$
**I:**	-0.256	-0.007	0.249	-0.131	-0.174	0.082	-0.201	-0.227
**II:**	-0.588				-0.494	0.094	-0.521	-0.559

[Cu(HAcbDM)(NO₃)] (**3**)

**$E_{initial}$:**	0.900 V	**E_{vertex}:**	-2.199 V	**Speed:**	0.200 V/s
**E_{final}:**	0.900 V	**Delay:**	0 s		

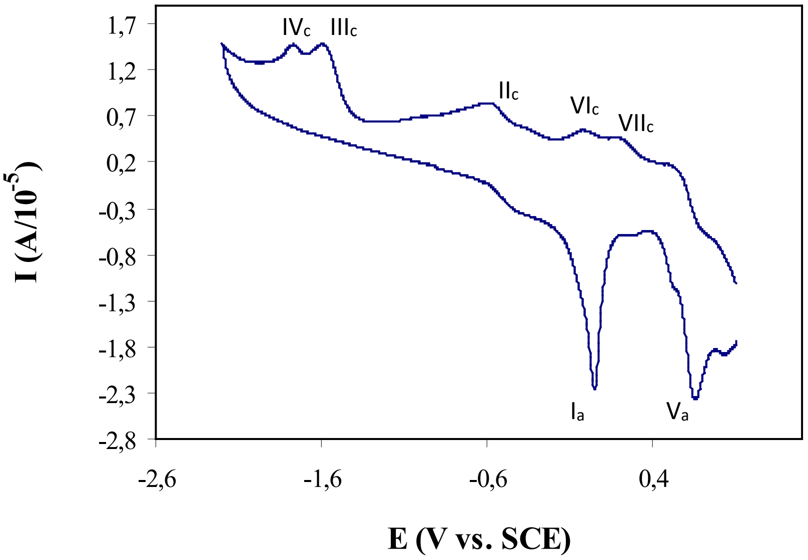

Peak Potentials

System	$(E_p)_c$	$(E_p)_a$	$(E_p)_a - (E_p)_c$	$[(E_p)_a + (E_p)_c]/2$	$(E_{p/2})_c$	$(E_{p/2})_c - (E_p)_c$	$(E_{1/2})_c$	$E^{o\prime}$
**I:**		0.048						
**II:**	-0.597				-0.465	0.132	-0.493	-0.569
**III:**	-1.593				-1.497	0.096	-1.525	-1.565
**IV:**	-1.77				-1.71	0.060	-1.738	-1.742
**V:**		0.651						
**VI:**	0.20				0.282	0.081	0.254	0.230
**VII:**	-0.036				0.045	0.081	0.017	-0.007

[Cu(HAcbDM)(NO$_3$)] (**3**)

**$E_{initial}$:**	0.400 V	**E_{vertex}:**	-1.300 V	**Speed:**	0.200 V/s
**E_{final}:**	0.400 V	**Delay:**	0 s		

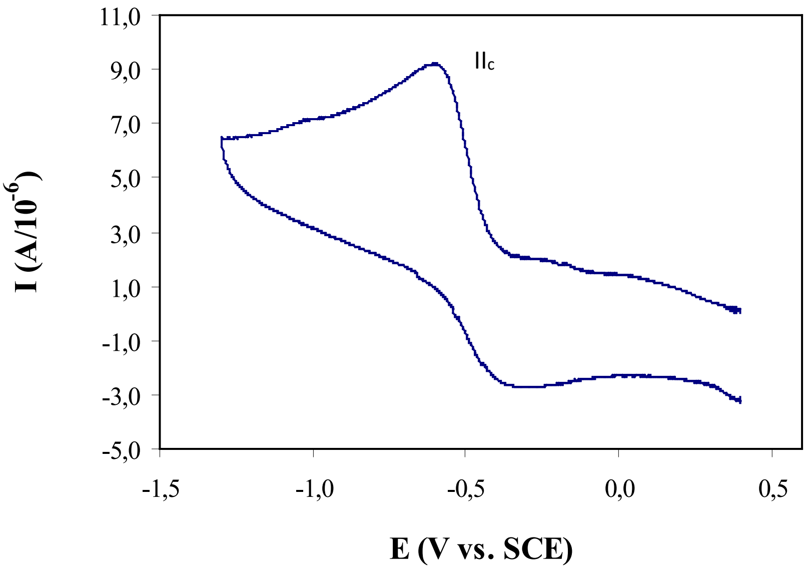

Peak Potentials

System	$(E_p)_c$	$(E_p)_a$	$(E_p)_a - (E_p)_c$	$[(E_p)_a + (E_p)_c]/2$	$(E_{p/2})_c$	$(E_{p/2})_c - (E_p)_c$	$(E_{1/2})_c$	$E^{o\prime}$
**II:**	-0.596				-0.492	0.104	-0.519	-0.567

[Cu(HAcbDM)(NO₃)] (**3**)

**$E_{initial}$:**	0.400 V	**E_{vertex}:**	-1.300 V	**Speed:**	0.200 V/s
**E_{final}:**	0.400 V	**Delay:**	10 s		

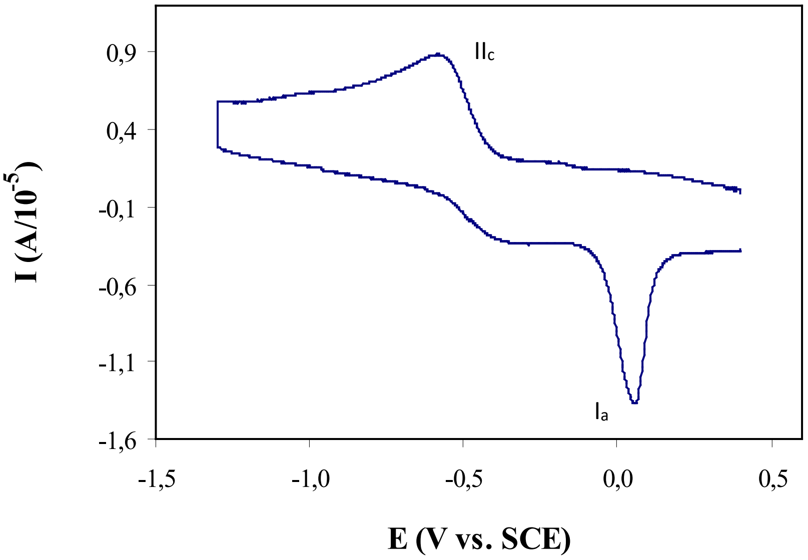

Peak Potentials

System	$(E_p)_c$	$(E_p)_a$	$(E_p)_a - (E_p)_c$	$[(E_p)_a + (E_p)_c]/2$	$(E_{p/2})_c$	$(E_{p/2})_c - (E_p)_c$	$(E_{1/2})_c$	$E^{o\prime}$
**I:**		0.057						
**II:**	-0.580				-0.482	0.098	-0.509	-0.551

[Cu(HAcbDM)(NO$_3$)] (**3**)

**E$_{initial}$:**	0.400 V	**E$_{vertex}$:**	-0.900 V	**Speed:**	0.200 V/s
**E$_{final}$:**	0.400 V	**Delay:**	10 s		

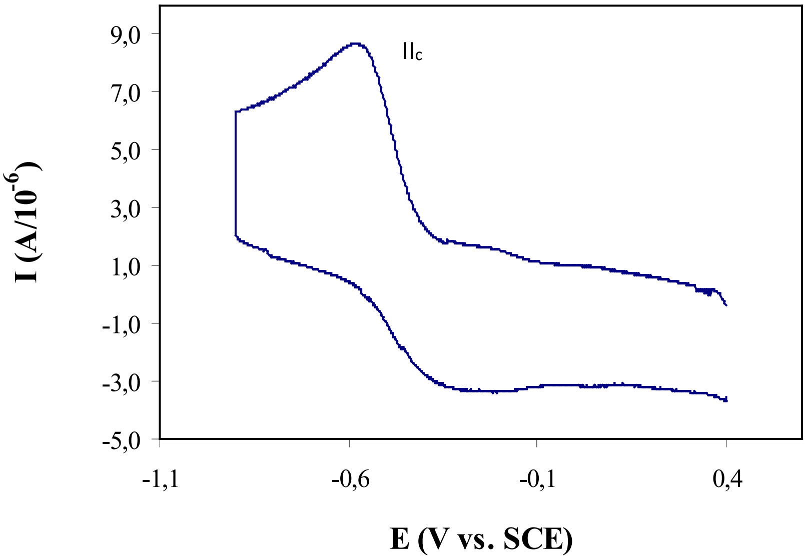

Peak Potentials

System	$(E_p)_c$	$(E_p)_a$	$(E_p)_a - (E_p)_c$	$[(E_p)_a + (E_p)_c]/2$	$(E_{p/2})_c$	$(E_{p/2})_c - (E_p)_c$	$(E_{1/2})_c$	$E^{o\prime}$
**II:**	-0.580				-0.478	0.102	-0.505	-0.551

[Cu(HAcbHexim)Cl]·1/2EtOH (**6**·1/2EtOH)

**$E_{initial}$:**	0.400 V	**E_{vertex}:**	-2.200 V	**Speed:**	0.020 V/s
**E_{final}:**	0.400 V	**Delay:**	0 s		

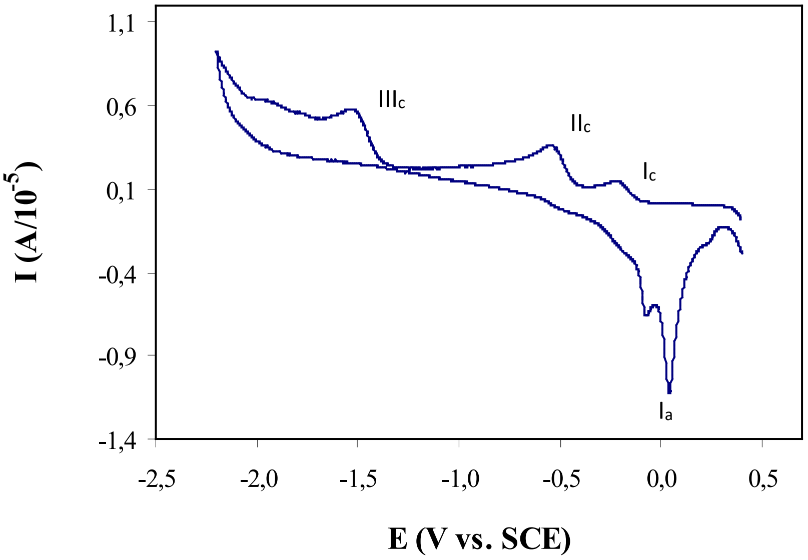

Peak Potentials

System	$(E_p)_c$	$(E_p)_a$	$(E_p)_a - (E_p)_c$	$[(E_p)_a + (E_p)_c]/2$	$(E_{p/2})_c$	$(E_{p/2})_c - (E_p)_c$	$(E_{1/2})_c$	$E^{o\prime}$
**I:**	-0.216	0.046	0.262	-0.085	-0.156	0.060	-0.184	-0.188
**II:**	-0.540				-0.476	0.064	-0.504	-0.511
**III:**	-1.546				-1.444	0.102	-1.472	-1.518

[Cu(HAcbHexim)Cl]·1/2EtOH (**6**·1/2EtOH)

**$E_{initial}$:**	0.400 V	**E_{vertex}:**	-1.200 V	**Speed:**	0.200 V/s
**E_{final}:**	0.400 V	**Delay:**	0 s		

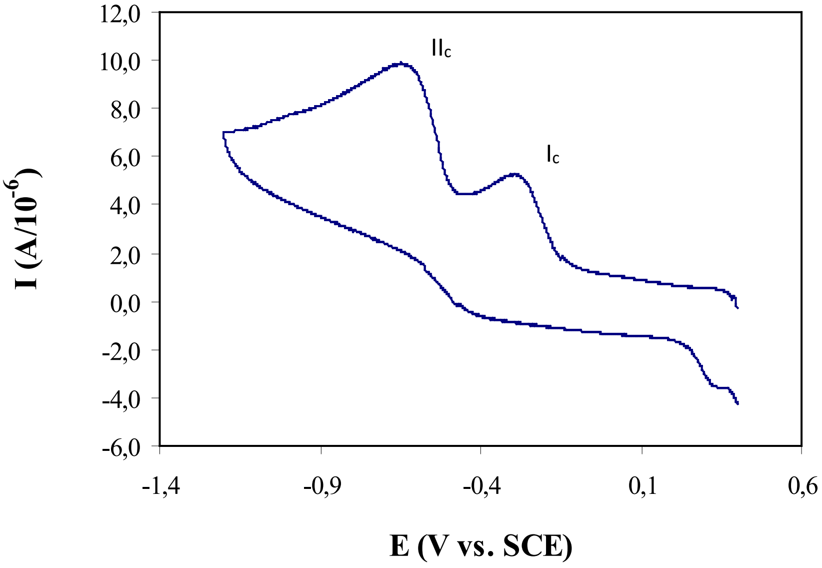

Peak Potentials

System	$(E_p)_c$	$(E_p)_a$	$(E_p)_a - (E_p)_c$	$[(E_p)_a + (E_p)_c]/2$	$(E_{p/2})_c$	$(E_{p/2})_c - (E_p)_c$	$(E_{1/2})_c$	$E^{o\prime}$
**I:**	-0.294				-0.206	0.088	-0.233	-0.265
**II:**	-0.646				-0.544	0.102	-0.571	-0.617

[Cu(HAcbHexim)Cl]·1/2EtOH (**6**·1/2EtOH)

**$E_{initial}$:**	0.400 V	**E_{vertex}:**	-0.800 V	**Speed:**	0.200 V/s
**E_{final}:**	0.400 V	**Delay:**	0 s		

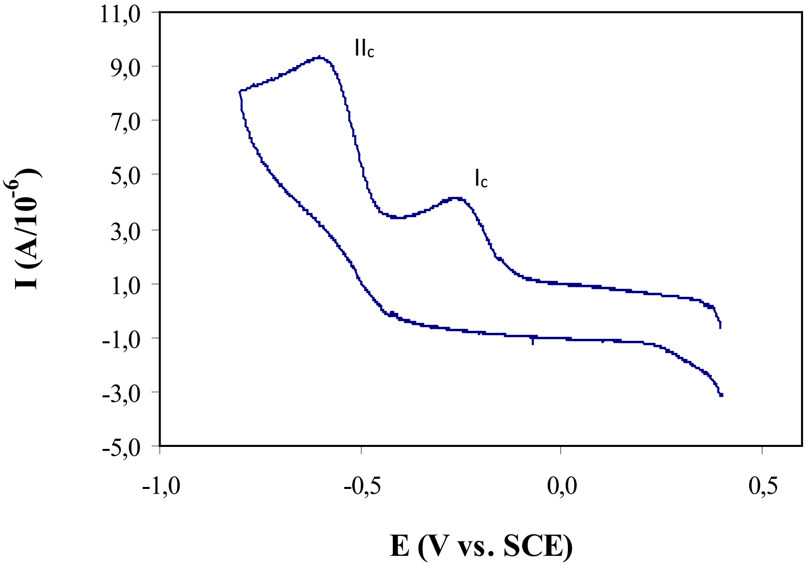

Peak Potentials

System	$(E_p)_c$	$(E_p)_a$	$(E_p)_a$ - $(E_p)_c$	$[(E_p)_a + (E_p)_c]/2$	$(E_{p/2})_c$	$(E_{p/2})_c$ - $(E_p)_c$	$(E_{1/2})_c$	$E^{o'}$
**I:**	-0.250				-0.184	0.066	-0.211	-0.221
**II:**	-0.604				-0.516	0.088	-0.543	-0.575

[Cu(HAcbHexim)Cl]·1/2EtOH (**6**·1/2EtOH)

**$E_{initial}$:**	0.400 V	**E_{vertex}:**	-0.800 V	**Speed:**	0.200 V/s
**E_{final}:**	0.400 V	**Delay:**	10 s		

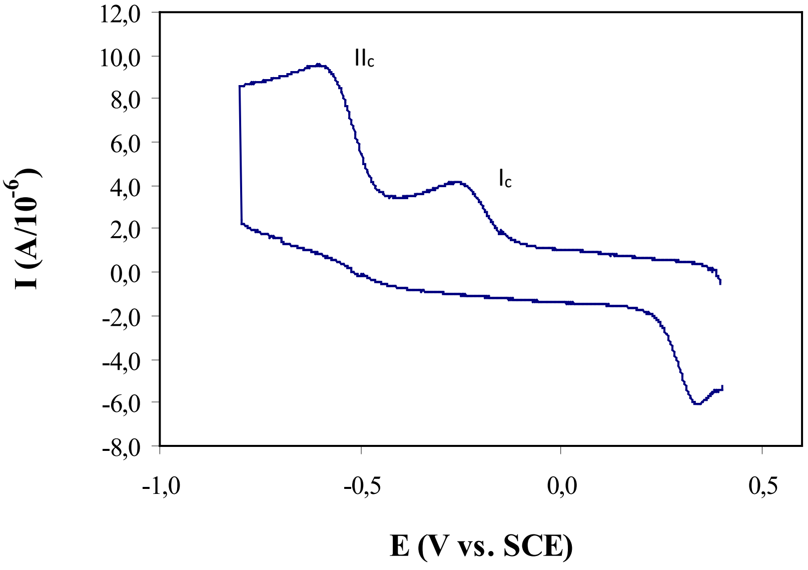

Peak Potentials

System	$(E_p)_c$	$(E_p)_a$	$(E_p)_a - (E_p)_c$	$[(E_p)_a + (E_p)_c]/2$	$(E_{p/2})_c$	$(E_{p/2})_c - (E_p)_c$	$(E_{1/2})_c$	$E^{o\prime}$
**I:**	-0.260				-0.186	0.074	-0.213	-0.231
**II:**	-0.610				-0.516	0.094	-0.543	-0.581

[Cu(HAcbHexim)Cl]·1/2EtOH (**6**·1/2EtOH)

**$E_{initial}$:**	0.400 V	**E_{vertex}:**	-0.400 V	**Speed:**	0.200 V/s
**E_{final}:**	0.400 V	**Delay:**	10 s		

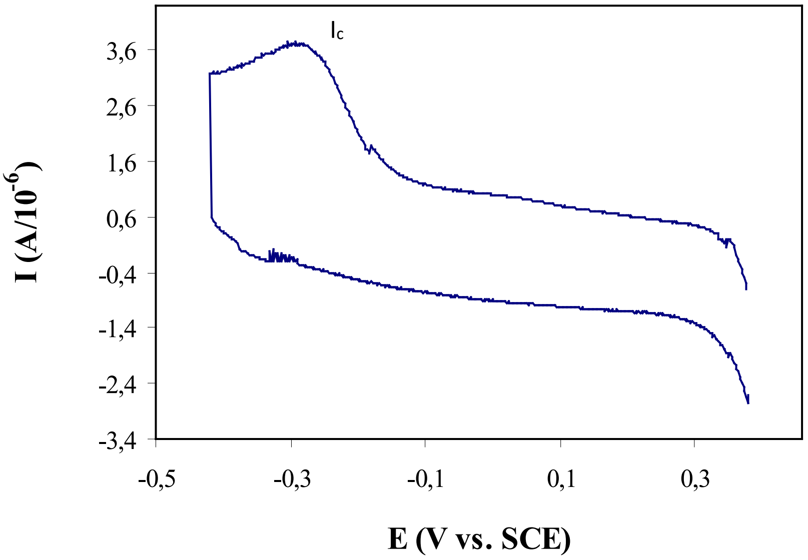

Peak Potentials

System	$(E_p)_c$	$(E_p)_a$	$(E_p)_a - (E_p)_c$	$[(E_p)_a + (E_p)_c]/2$	$(E_{p/2})_c$	$(E_{p/2})_c - (E_p)_c$	$(E_{1/2})_c$	$E^{o'}$
**I:**	-0.272				-0.192	0.080	-0.219	-0.243

[Cu(HAcbHexim)(NO$_3$)]·1/2H$_2$O (**7**·1/2H$_2$O)

**E$_{initial}$:**	0.900 V	**E$_{vertex}$:**	-2.199 V	**Speed:**	0.200 V/s
**E$_{final}$:**	0.900 V	**Delay:**	0 s		

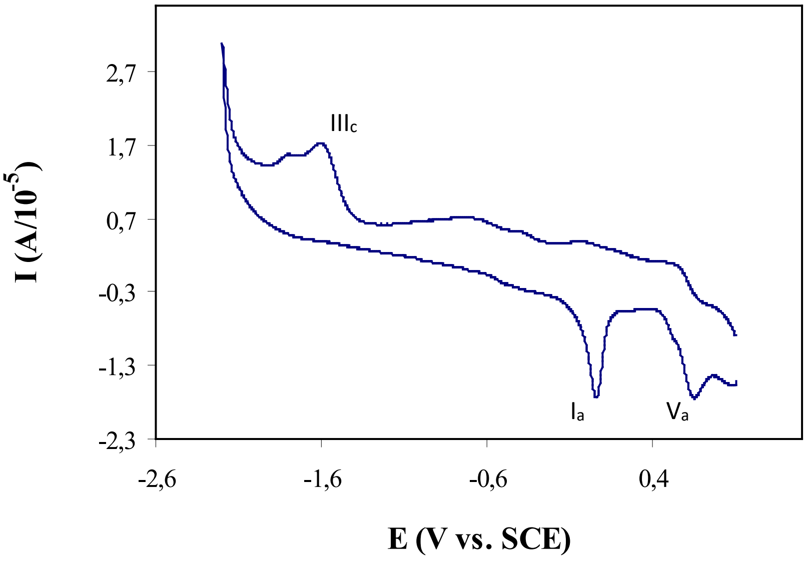

Peak Potentials

System	(E$_p$)$_c$	(E$_p$)$_a$	(E$_p$)$_a$ - (E$_p$)$_c$	[(E$_p$)$_a$ + (E$_p$)$_c$]/2	(E$_{p/2}$)$_c$	(E$_{p/2}$)$_c$ - (E$_p$)$_c$	(E$_{1/2}$)$_c$	Eº'
**I:**		0.060						
**III:**	-1.602				-1.497	0.105	-1.525	-1.573
**V:**		0.651						

[Cu(HAcbHexim)(NO$_3$)]·1/2H$_2$O (**7**·1/2H$_2$O)

**$E_{initial}$:**	0.400 V	**E_{vertex}:**	-1.100 V	**Speed:**	0.200 V/s
**E_{final}:**	0.898 V	**Delay:**	0 s		

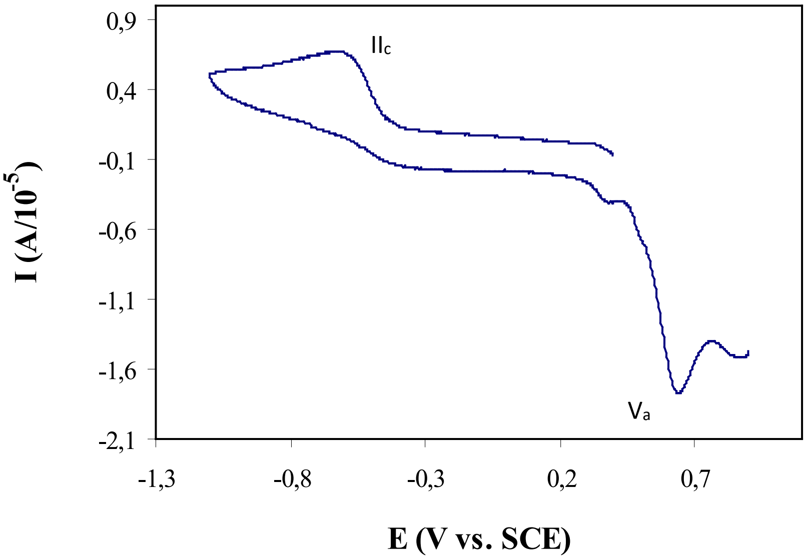

Peak Potentials

System	$(E_p)_c$	$(E_p)_a$	$(E_p)_a - (E_p)_c$	$[(E_p)_a + (E_p)_c]/2$	$(E_{p/2})_c$	$(E_{p/2})_c - (E_p)_c$	$(E_{1/2})_c$	$E^{o\prime}$
**II:**	-0.620				-0.500	0.120	-0.528	-0.591
**V:**		0.643						

[Cu(HAcbHexim)(NO$_3$)]·1/2H$_2$O (**7**·1/2H$_2$O)

**E$_{initial}$:**	0.400 V	**E$_{vertex}$:**	-1.100 V	**Speed:**	0.200 V/s
**E$_{final}$:**	0.898 V	**Delay:**	10 s		

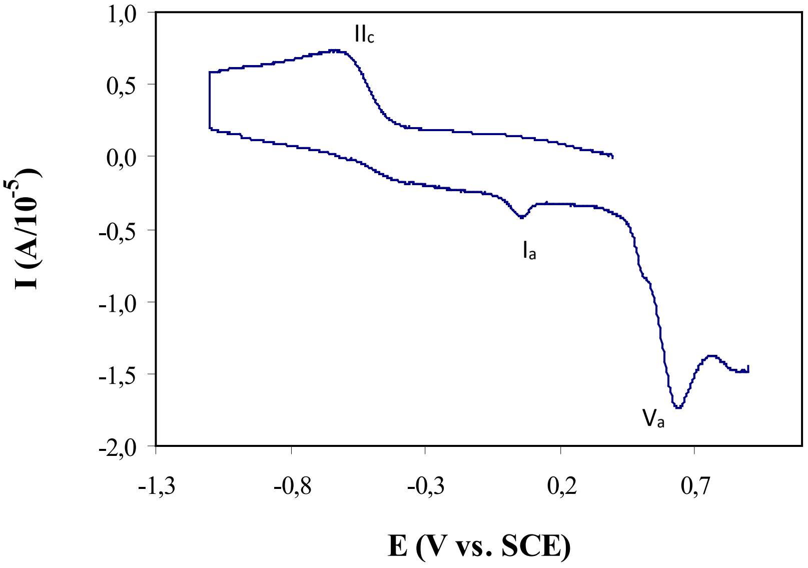

Peak Potentials

System	(E$_p$)$_c$	(E$_p$)$_a$	(E$_p$)$_a$ - (E$_p$)$_c$	[(E$_p$)$_a$ + (E$_p$)$_c$]/2	(E$_{p/2}$)$_c$	(E$_{p/2}$)$_c$ - (E$_p$)$_c$	(E$_{1/2}$)$_c$	Eº'
**I:**		0.058						
**II:**	-0.632				-0.506	0.126	-0.534	-0.603
**V:**		0.643						

[Cu(HAcbHexim)(NO3)]·1/2H2O (**7**·1/2H2O)

**$E_{initial}$:**	0.200 V	**E_{vertex}:**	-1.300 V	**Speed:**	0.200 V/s
**E_{final}:**	0.896 V	**Delay:**	10 s		

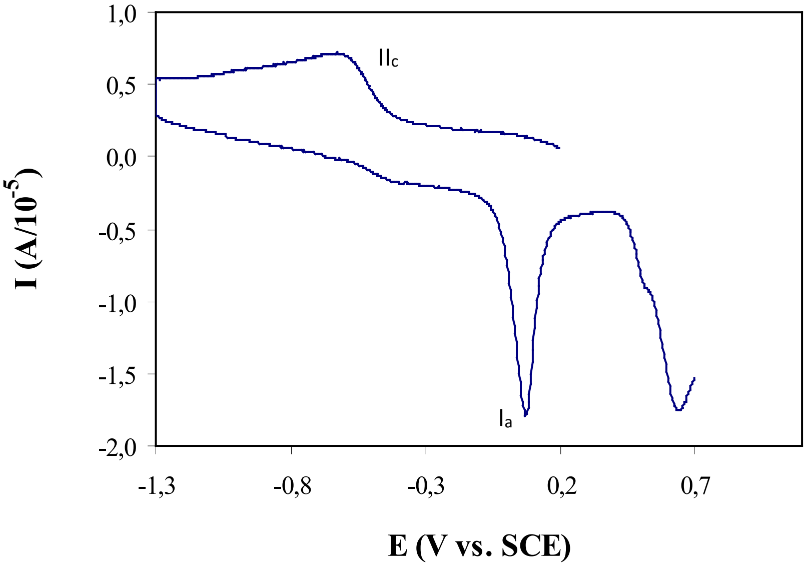

Peak Potentials

System	$(E_p)_c$	$(E_p)_a$	$(E_p)_a - (E_p)_c$	$[(E_p)_a + (E_p)_c]/2$	$(E_{p/2})_c$	$(E_{p/2})_c - (E_p)_c$	$(E_{1/2})_c$	Eº'
**I:**		0.074						
**II:**	-0.628				-0.502	0.126	-0.530	-0.599

[Cu(HAcbHexim)ClO4] (**8**)

**$E_{initial}$:**	0.900 V	**E_{vertex}:**	-2.200 V	**Speed:**	0.020 V/s
**E_{final}:**	0.900 V	**Delay:**	0 s		

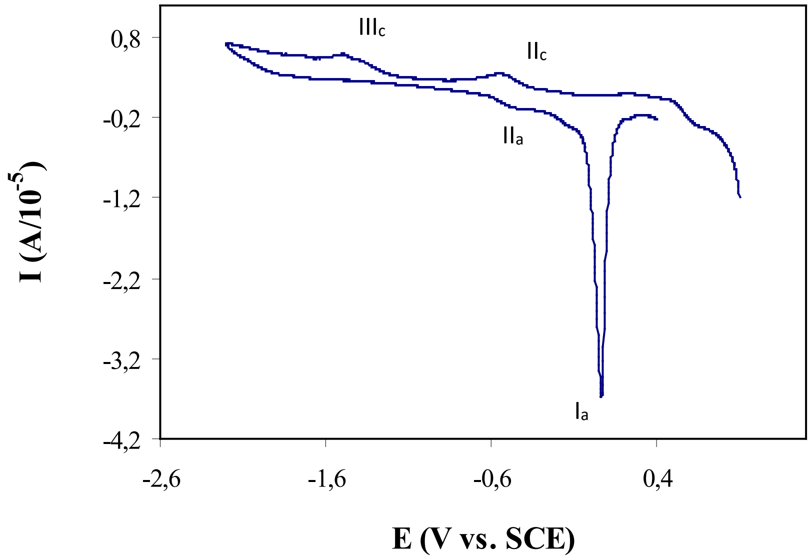

Peak Potentials

System	$(E_p)_c$	$(E_p)_a$	$(E_p)_a - (E_p)_c$	$[(E_p)_a + (E_p)_c]/2$	$(E_{p/2})_c$	$(E_{p/2})_c - (E_p)_c$	$(E_{1/2})_c$	Eº'
**I:**		0.066						
**II:**	-0.55	-0.428	0.122	-0.489	-0.94	-0.390	-0.968	-0.522
**III:**	-1.492				-1.322	0.170	-1.350	-1.464

[Cu(HAcbHexim)ClO$_4$] (**8**)

**E$_{initial}$:**	0.400 V	**E$_{vertex}$:**	-1.300 V	**Speed:**	0.200 V/s
**E$_{final}$:**	0.400 V	**Delay:**	0 s		

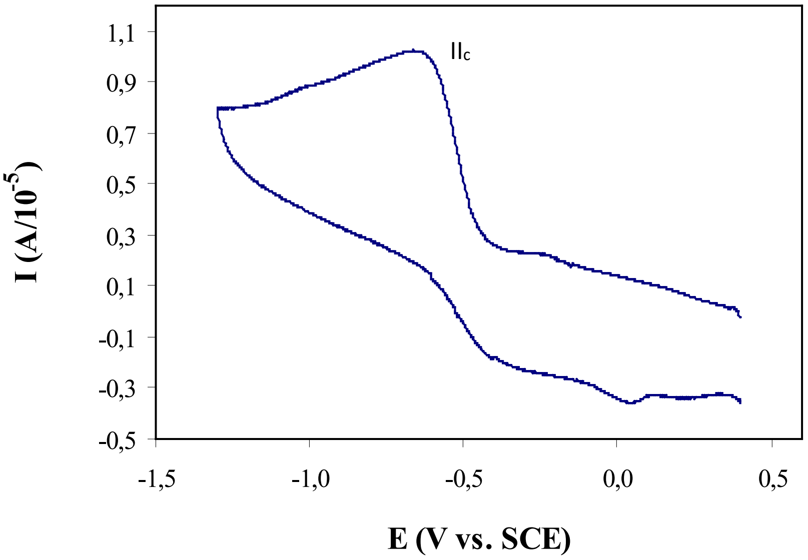

Peak Potentials

System	(Ep)c	(Ep)a	(Ep)a - (Ep)c	[(Ep)a + (Ep)c]/2	(Ep/2)c	(Ep/2)c - (Ep)c	(E1/2)c	Eº'
**II:**	-0.662				-0.520	0.142	-0.547	-0.633

[Cu(HAcbHexim)ClO4] (**8**)

$E_{initial}$: 0.400 V **E_{vertex}:** -1.300 V **Speed:** 0.200 V/s
E_{final}: 0.400 V **Delay:** 10 s

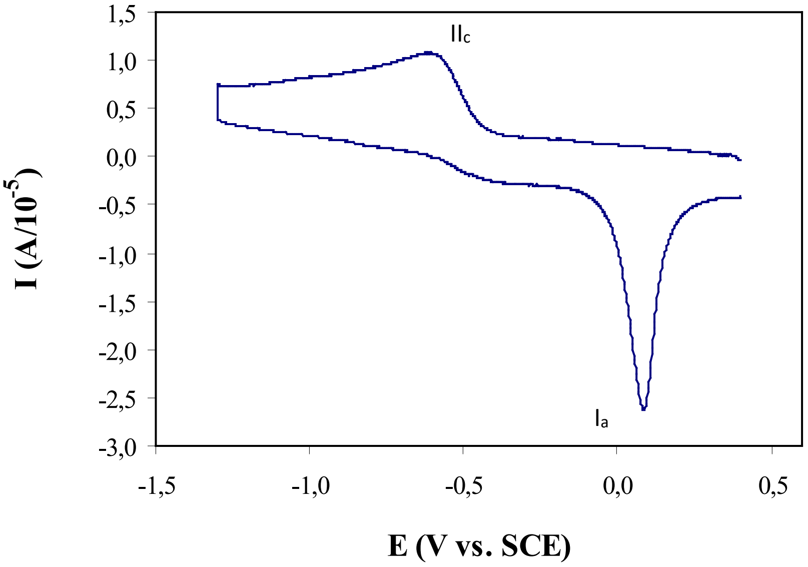

Peak Potentials

System	$(E_p)_c$	$(E_p)_a$	$(E_p)_a - (E_p)_c$	$[(E_p)_a + (E_p)_c]/2$	$(E_{p/2})_c$	$(E_{p/2})_c - (E_p)_c$	$(E_{1/2})_c$	$E^{o\prime}$
**I:**		**0.085**						
**II:**	**-0.604**				**-0.506**	0.098	-0.534	-0.576

[Cu(HAcbHexim)ClO4] (**8**)

**$E_{initial}$:**	0.400 V	**E_{vertex}:**	-0.900 V	**Speed:**	0.200 V/s
**E_{final}:**	0.400 V	**Delay:**	0 s		

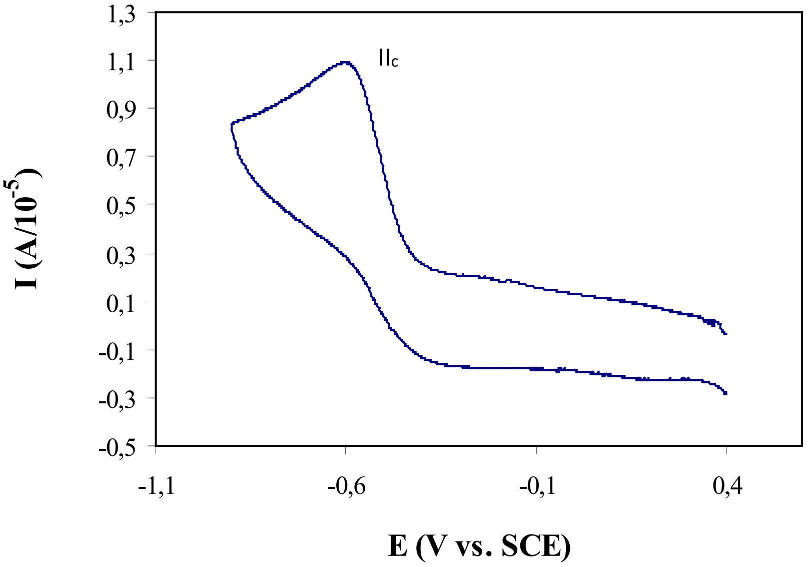

Peak Potentials

System	$(E_p)_c$	$(E_p)_a$	$(E_p)_a - (E_p)_c$	$[(E_p)_a + (E_p)_c]/2$	$(E_{p/2})_c$	$(E_{p/2})_c - (E_p)_c$	$(E_{1/2})_c$	$E^{o\prime}$
**II:**	-0.604				-0.502	0.102	-0.529	-0.575
